# T cell receptor (TCR) signaling in health and disease

**DOI:** 10.1038/s41392-021-00823-w

**Published:** 2021-12-13

**Authors:** Kinjal Shah, Amr Al-Haidari, Jianmin Sun, Julhash U. Kazi

**Affiliations:** 1 Division of Translational Cancer Research, Department of Laboratory Medicine, Lund University, Lund, Sweden; 2 Lund Stem Cell Center, Department of Laboratory Medicine, Lund University, Lund, Sweden; 3 Clinical Genetics and Pathology, Skåne University Hospital, Region Skåne, Lund, Sweden; 4 Clinical Sciences Department, Surgery Research Unit, Lund University, Malmö, Sweden; 5 NHC Key Laboratory of Metabolic Cardiovascular Diseases Research, Science and Technology center, School of Basic Medical Sciences, Ningxia Medical University, Yinchuan, China

**Keywords:** Haematological cancer, Lymphocytes

## Abstract

Interaction of the T cell receptor (TCR) with an MHC-antigenic peptide complex results in changes at the molecular and cellular levels in T cells. The outside environmental cues are translated into various signal transduction pathways within the cell, which mediate the activation of various genes with the help of specific transcription factors. These signaling networks propagate with the help of various effector enzymes, such as kinases, phosphatases, and phospholipases. Integration of these disparate signal transduction pathways is done with the help of adaptor proteins that are non-enzymatic in function and that serve as a scaffold for various protein–protein interactions. This process aids in connecting the proximal to distal signaling pathways, thereby contributing to the full activation of T cells. This review provides a comprehensive snapshot of the various molecules involved in regulating T cell receptor signaling, covering both enzymes and adaptors, and will discuss their role in human disease.

## Introduction

T cells are key mediators in mounting an effective adaptive cell-mediated immune response.^1,2^ T cells continuously screen lymphoid and peripheral tissues for antigens such as peptides or lipids displayed by major histocompatibility complex (pMHC) molecules of other cells. Normal T cell development in the thymus undergoes a major developmental checkpoint in which T cell receptor (TCR) signaling is involved. Thymocytes bearing TCR with a high affinity for self-peptide MHC complexes undergo apoptosis (negative selection), whereas those bearing low-affinity TCR survive and differentiate into mature T cells (positive selection).^3^ This ensures that only those T cells that are self-tolerant survive while eliminating the self-reactive T cells.^4^ These naïve single positive mature T cells then leave the thymus and enter the peripheral lymphoid organs, such as the spleen and lymph nodes, where they get exposed to foreign peptides presented by the MHC molecules of antigen-presenting cells (APCs), such as the macrophages, dendritic cells, and B cells, during pathogenic infection.^5^ Upon engagement of TCR with the antigenic peptide, T cells get activated, undergoing clonal expansion and differentiation to perform their effector functions, due to a complex series of molecular changes at the plasma membrane, cytoplasm, and nucleus.^6,7^ T cell signaling is thus important for efficient T cell development, activation, and immune tolerance. TCR signaling dysregulation can thus lead to anergy or autoimmunity.^8^

In general, the transmission of external cues to the interior of the cell occurs through binding of a ligand to the extracellular domain of the receptor, leading to receptor aggregation or conformational changes. Once this is accomplished, protein tyrosine kinases (PTKs) phosphorylate various tyrosine residues present in the cytoplasmic tail of the receptor, which serve as a docking site for various signaling molecules containing specific phosphotyrosine recognition domains such as the SRC homology 2 (SH2) and phosphotyrosine-binding (PTB) domains.^6^ This initiates proximal biochemical signals mediated by the key effector enzymes, such as kinases, phosphatases, and phospholipases, that culminate in distal signaling by activating numerous transcription factors required for translating these events into gene activation.^9^ However, both the proximal and distal signaling need to be integrated, and this is done by various adaptor proteins whose main function is to form multiprotein complexes.^10^

Adaptors are proteins that usually lack intrinsic enzymatic activity and instead possess multiple binding domains for phosphotyrosine, proline-rich region and lipid interactions, and sequence motifs that in turn are involved in binding to such domains.^11,12^ The phosphorylated tyrosine residues on various adaptor proteins serve as binding sites for many critical effector enzymes and other adaptor proteins.^6^ Thus, they behave as scaffolds, facilitating protein–protein interactions that aid in forming multiprotein complexes, thereby integrating signaling cascades necessary for efficient T cell biology. Apart from this, adaptors also interact with other adaptors present at the plasma membrane microdomains and even play important roles in the regulation of the cytoskeleton.^6,10,13^ Hematopoietic-specific adaptor proteins have led to a better understanding of T cell signaling.^13^ Those adaptor proteins can regulate signal transduction both positively and negatively.^10,13^ In this review, we discuss the role of TCR signaling in human health and disease.

## Components and structure of TCR complex

The core TCR complex consists of two TCR chains and six cluster of differentiation 3 (CD3) chains. Several other components include coreceptors, kinases, and ligands.^14,15^

### TCR-CD3 chains

The human genome expresses four TCR genes known as TCRα, TCRβ, TCRγ, and TCRδ, which forms two distinct heterodimers: TCRα/TCRβ or TCRγ/TCRδ.^16–18^ The majority of mature T cells expresses TCRα and TCRβ isoforms, generally referred to as T cells (or αβ T cells), while a small portion (0.5–5%) of T lymphocytes (γδ T cells) expresses TCRγ and TCRδ isoforms.^19^ In this review, we will focus on αβ T cells, and henceforth the nomenclature T cells will refer to αβ T cells.

Both heterodimers form multiprotein complexes with CD3 δ, γ, ε, and ζ chains. TCR chains consist of an extracellular region, transmembrane region, and a shorter cytoplasmic tail. The extracellular region contains a variable immunoglobulin-like (V) domain, a constant immunoglobulin-like (C) domain, and connecting peptide.^20^ The RAG1 and RAG2 recombinases facilitate the assembly of the V domain from gene segments that serve as the antigen recognition site. The C domain is used for the interactions with CD3 chains.

**Fig. 1:**
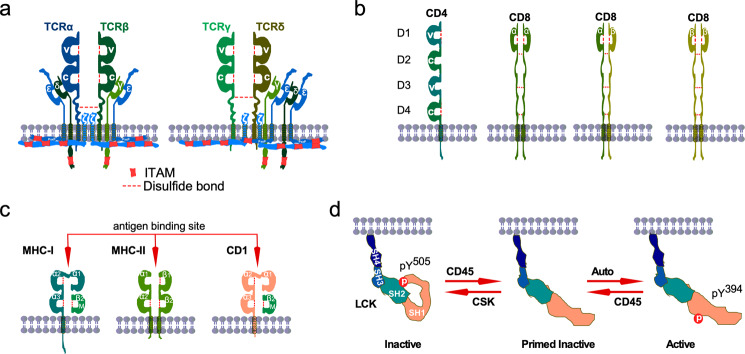
TCR components. **a** TCRα/TCRβ and TCRγ/TCRδ heterodimers form complexes with the CD3 molecules. Heterodimers of CD3ε/CD3δ and CD3γ/CD3ε, and a homodimer of CD3ζ/CD3ζ form complexes with TCR dimers. TCR heterodimers contain intramolecular and intermolecular disulfide bonds. CD3 chains contain 10 ITAMs distributed in different CD3 molecules. The variable region (V) of TCR heterodimers recognize the antigen peptide-loaded on MHC (pMHC). In the absence of pMHC, the intracellular part of the CD3 molecules forms a close conformation in which ITAMs are inaccessible to the kinases for phosphorylation. **b** Coreceptor CD4 acts as a single molecule while CD8α and CD8β can form homodimers or heterodimers. **c** MCH-I consists of an α-chain containing three immunoglobulin domains (α_1_, α_2_, α_3_) and β2-microglobulin (β2m). MCH-2 is the heterodimer of an α chain and a β-chain containing two immunoglobulin domains (α_1_, α_2,_ and β_1_, β_2_) in each chain. **d** LCK-loaded CD4 molecules bind to the MHC-II bound TCR (TCRα/TCRβ) complex. This allows LCK to phosphorylate two distinct sites on ITAMs. Then ZAP-70 interacts with the phosphotyrosine sites and mediates more tyrosine phosphorylation. CD4 and MHC-II interaction is mediated through the membrane-proximal α_2_ and β_2_ domains of MHC-II and the membrane-distal D1 domain of CD4.

There are considerable structural differences between αβ and γδ chains in terms of C domain and connecting peptide, which are also reflected in the assembly of the TCR complexes, surface shape, and charge distribution.^21–24^ However, in both complexes, three dimers of CD3 proteins, δε and γε heterodimers and ζζ homodimers, are present.^23,25^ These CD3 proteins associate with TCR via non-covalent hydrophobic interactions and are required for a complete TCR localization on the cell surface (Fig. 1a).

### TCR co-receptors

Initial studies demonstrated that T cells expressing common TCRα/TCRβ heterodimer with distinct functions—for example, cytotoxic T cells that directly destroy infected cells and a subset of helper T cells that help B cells—may easily be distinguishable by the expression of mutually exclusive cell surface molecules CD8 and CD4.^26–30^ Later studies indicated that these two receptors may play important roles in the association of MHC molecules and thus are referred to as co-receptors.^31–33^ Both CD4 and CD8 molecules play important roles during the development of T cells by helping the TCR complex select a different class of MHC molecules.^34^ Like TCR molecules, both CD4 and CD8 molecules contain an extracellular domain, transmembrane domain, and a short intracellular tail.^35^ Although both CD8 and CD4 act as coreceptors with similar functionality, they share a minimal structural similarity. The extracellular domain of CD4 contains two V domains (D1 and D3) and two C domains (Fig. 1b). CD4 acts as a monomer on the T cell surface where it uses the D1 domain for MHC recognition and the cytoplasmic tail for interaction with non-receptor tyrosine kinase LCK.^36^ On the other hand, the CD8 extracellular domain contains only a single V domain. However, two CD8 isoforms, CD8α and CD8β, are expressed and can form homo- or heterodimers (Fig. 1b). Most CD8-positive T cells express as a heterodimer; some CD8-positive T cells—for example, intraepithelial lymphocytes and memory precursors—express as an αα homodimer.^37–39^

### TCR ligands

Ligands for T cells are divided into two classes: MHC class I (MHCI) and MHC class II (MHCII) (Fig. 1c). Human MHCIs are complexes of human leukocyte antigens (HLAs: HLA-A, HLA-B, and HLA-C) and β2-microglobulin while MHCIIs are heterodimers of several HLAs (HLA-DP, HLA-DQ, and HLA-DR).^40^ Antigen peptide-bound MHCI (pMHC-I) molecules can be presented on any nucleated cells recognized by CD8+ T cells. On the other hand, CD4+ T cells recognize antigen peptide-bound MHCII (pMHC-II) molecules that are presented on the APCs, such as B cells, macrophages, and dendritic cells.^40^ Besides peptide presentation by MHC molecules, lipid antigens present similarly structurally to MHCII molecules, such as CD1 family proteins.^41,42^

### Lymphocyte-specific PTK (LCK)

LCK is a member of the SRC family kinase (SFK). The SFKs are a family of ten structurally similar non-receptor PTKs which have been implicated in various cellular functions.^43–45^ All SFKs contain highly conserved regulatory domains (SH3 and SH2), a protein tyrosine kinase domain (SH1), a C-terminal tail, and a poorly conserved N-terminal region (SH4 domain). The SH4 domain contains a myristoylation site by which SFKs anchor to the membrane.^46^ The SH3 domain, in general, recognizes proline-rich motifs (PxxP), and the SH2 domain interacts with phosphotyrosine residues.^47,48^ However, the function of the SH4 domain cannot be generalized for all SFKs except that it holds the myristoylation site. The SH4 domain of some SFKs also contains a palmitoylation in addition to the myristoylation site. Likewise, other SFKs’ LCK activity is tightly controlled by phosphorylation/dephosphorylation cycles (Fig. 1d). C-terminal SRC kinase (CSK) phosphorylates LCK on Y^505^ residue that interacts with the LCK-SH2 domain, keeping it inactive.^49^ The leukocyte common antigen (CD45), also known as protein tyrosine phosphatase receptor type C (PTPRC), removes the regulatory phosphotyrosine residue that releases the kinase domain from autoinhibitory states (priming) and also makes the SH2 domain available for interaction with other proteins.^50–52^ This priming step results in autophosphorylation of Y,^394^ leading to full activation of LCK.^52^ Besides a role in LCK activation, several studies have pointed out that CD45 can negatively regulate LCK function by removing tyrosine phosphorylation from LCK-Y^394^.^53,54^ T cells maintaining a certain level of CD45 expression uphold appropriate LCK activation whereas low levels of CD45 expression correlate with the lower TCR activation, and higher CD45 expression reduces LCK activity by removing tyrosine phosphorylation from LCK-Y^394^.^53^ Thus, T cells can regulate TCR activity by modulating CD45 expression.^55^ Several cytosolic phosphatases, including PTPN6(SHP-1), and PTPN22, also control LCK activity by removing phosphate group from Y^394^.^56,57^ Therefore, LCK activity is probably dynamically regulated by cellular abundance and activity of CSK, CD45, PTPN6, and PTPN22.

## TCR activation and proximal signaling

Early T cell signaling takes place within a few seconds, and the first step is TCR activation.^58^ An early event in the proximal signaling of TCR is the involvement and activation of a set of PTKs.^6^ Several PTKs, such as LCK, FYN, and ZAP-70, are important signaling components for T cell development and activation of TCR signaling through tyrosine phosphorylation on CD3.^8,59–61^ The cytosolic tail of the CD3 proteins contains a unique motif, the immunoreceptor tyrosine-based activation motifs (ITAMs), that consists of two tyrosine residues flanked by leucine/isoleucine and spaced by bulky aromatic amino acid, thus having a consensus sequence of D/Ex_(0-2)_YxxI/Lx_(6-8)_YxxI/L.^62–64^ Each of the CD3δ, γ, and ε chains contain one ITAM each, whereas each CD3ζ chains contain three ITAMs, thus each TCR-CD3 complex contains ten ITAMs.^6,65,66^ For TCR activation, tyrosine residues in ITAMs need to be phosphorylated, which is initiated by LCK and, to some degree, by FYN.^67–69^ Although FYN can induce phosphorylation of ITAMs, its role is dispensable for T cell development.^70,71^ Thus, tyrosine phosphorylation on CD3 ITAMs by LCK during T cell development probably cannot be replaced by other tyrosine kinases.^72^

LCK is known to be associated with several growth factor receptors, including KIT, FLT3, and AXL, in a phosphorylation-dependent manner.^73–75^ The interaction between LCK and growth factor receptors is mediated via the SH2 domain of LCK that interacts with the phosphotyrosine residue of the activated receptor. Since the TCR-CD3 complex lacks intrinsic kinase activity, the pMHC-loaded TCR-CD3 complex remains unphosphorylated, and therefore LCK cannot directly interact with the inactive complex through an SH2 domain. However, to facilitate the phosphorylation of ITAMs, LCK needs to be localized to the cell membrane. LCK can anchor to the cell membrane via its myristoylation (serine 2) and palmitoylation (cysteine 3/5) sites present in the SH4 domain or through the interaction with the cytoplasmic tail of coreceptors CD4 and CD8.^69,76–88^ The C-terminal tail of CD4 and CD8α contains a conserved CxCP motif, which is absent in CD8β, required for this interaction.^81,89,90^ This motif interacts with the CxxC motif present in the LCK SH4 domain, mediating the interaction in a zinc-ion-dependent manner.^89–94^ Therefore, only the homodimer CD8α/CD8α and heterodimeric CD8α/CD8β can load LCK to the TCR complex, and although a CD8β/CD8β homodimer can be formed, it cannot recruit LCK to the TCR complex and thereby does not play a role in TCR signaling.

**Fig. 2:**
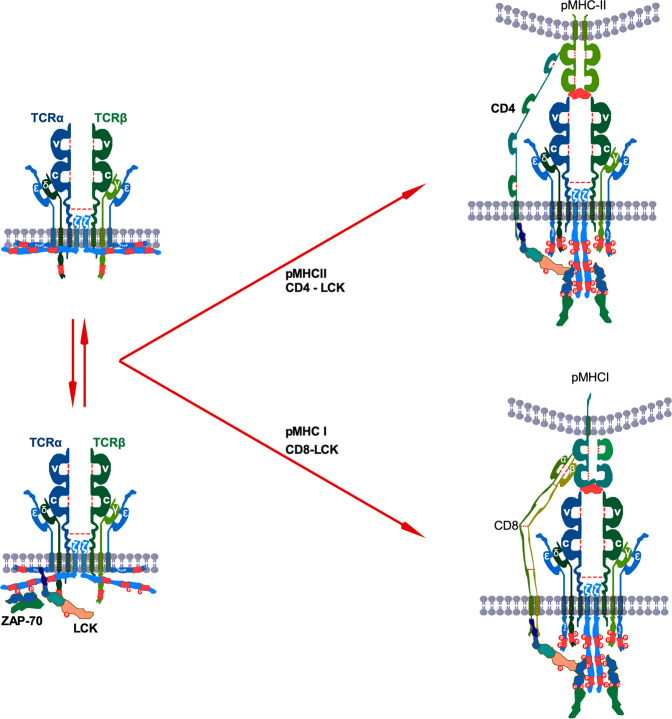
TCR activation. In resting T cells, CD3ζ and CD3ε remain membrane-embedded. Perhaps membrane-bound CD3ζ might be released to the cytosol, where free LCK induces tyrosine phosphorylation on at least two sites in ITAMs. This basal tyrosine phosphorylation creates docking sites for ZAP-70 interaction. After antigen engagement, the TCR complex recruits coreceptor-bound LCK that phosphorylates ZAP-70 and interacts with it through the SH2 domain facilitating tyrosine phosphorylation on other residues on ITAMs.

T cells usually express CD4 or CD8 coreceptors, and therefore pMHC-bound TCR browses for LCK-loaded coreceptors where the non-polymorphic part of pMHCs interacts with the distal membrane part of the coreceptors.^76–79^ The membrane-distal D1 domain of CD4 associates with the membrane-proximal α_2_ and β_2_ domains of MHC-II (Fig. 2), but it does not directly interact with the TCR complex.^95–98^ Similar to the CD4–MHC-II interaction, binding with the CD8α/CD8α homodimer or CD8α/CD8β heterodimer to the MHC-I complex is mediated through the membrane-distal D1 domain of CD8 and membrane-proximal β2M and α3 of the MHCI complex.^99^ Additionally, the membrane distal α2 domain of the MHC-I complex also participates in interaction with CD8.^99^ Such interaction keeps TCR and coreceptors orthogonal, which is likely important for the stability of the complex, LCK loading, and TCR activation.^100–103^ Although both the CD8α/CD8α homodimer or CD8α/CD8β heterodimer binds with MHC-1 complex with a similar affinity, the CD8β/CD8β homodimer does not bind with MHC-1.^104–106^

Interaction between coreceptors and LCK has two important functions: it brings LCK in close proximity to the TCR complex and it stabilizes coreceptors by preventing clathrin-mediated endocytosis.^94^ However, once LCK phosphorylates CD3 proteins, it leaves coreceptors that result in the internalization of coreceptors.^107–111^ Nevertheless, the interaction between LCK-loaded coreceptor and pMHC acts as a rate-limiting step to initiate TCR signaling.^112^

Initial studies suggest that coreceptor-bound LCK mediates tyrosine phosphorylation on all four CD3 chains.^113–116^ However, those studies have not suggested any site-specificity or whether LCK loaded to the CD4 or CD8 makes any difference in CD3 tyrosine phosphorylation dynamics. To address the sequence of CD3 tyrosine phosphorylation, several attempts have been taken with a specific focus on CD3ζ, which has six tyrosine phosphorylation sites.^117,118^ In resting T cells, at least two tyrosine residues in second and third ITAMs of CD3ζ remain to be phosphorylated.^117^ When activated, N-terminal tyrosine residue in the third ITAM displays dependency on the N-terminal tyrosine residue of the first ITAM, and C-terminal tyrosine residue in the second ITAM needs the C-terminal tyrosine residue to be phosphorylated on the first ITAM.^117^ Furthermore, LCK phosphorylates all six tyrosine residues of recombinant CD3ζ ITAMs in a specific order, starting from the N-terminal tyrosine residue of the first ITAM.^118^ These studies suggest that tyrosine phosphorylation in ITAMs is a controlled molecular event that has further been shown to be regulated by TCR ligand.^117^

LCK-induced phosphorylation of both tyrosine residues in ITAM has been shown to be required for interaction with ZAP-70 where ZAP-70 SH2 domains mediate the interactions.^67,119^ This interaction stabilizes tyrosine phosphorylation of CD3ζ and induces ZAP-70 tyrosine phosphorylation, which was independent of ZAP-70 kinase activity, suggesting that ZAP-70 does not play a role in CD3ζ tyrosine phosphorylation but that interaction with tyrosine residues probably limits phosphatase access, protecting tyrosine phosphorylation. Nevertheless, the interaction is important for ZAP-70 activation and downstream signaling.

The mechanism through which CD3 ITAMs remain unphosphorylated in an inactive TCR complex remains to be debated. Using CD3ε as a model, it has been depicted that the positively charged cytoplasmic domain remains embedded in the negatively charged inner membrane, which sequestrates tyrosine residues.^120,121^ Similarly, the cytoplasmic part of CD3ζ remains lipid-bound, preventing tyrosine phosphorylation.^122^ However, the role of the inner membrane in the prevention of tyrosine phosphorylation was questioned by another study.^123^ This study demonstrated that removal of positively charged residues did not enhance tyrosine phosphorylation but that pervanadate enhanced CD3 tyrosine phosphorylation, concluding that phosphatase might be involved in the prevention of tyrosine phosphorylation.^123^ Pervanadate prevents tyrosine phosphatases by oxidizing the catalytic cysteine of phosphatase.^124^ Those claims were later contested by the fact that removal of positively charged residues decreases LCK-mediated CD3 tyrosine phosphorylation, and pervanadate can prevent membrane association of CD3 with lipid membranes.^125^

Combining early studies, a model has been proposed suggesting that interaction between the cytoplasmic chain and lipid membrane prevents ITAM phosphorylation, and pMHC association to the TCR induces structural changes that release CD3 from the inner membrane.^126,127^ This model has further been supported by the fact that positively charged ions, such as Ca^2+^, can release sequestered CD3 chains, facilitating tyrosine phosphorylation,^128^ and that defects in Mg^2+^ transport are linked to the defective T cell activation due to impaired Ca^2+^ influx in T cells.^129^ Antigen engagement increases Ca^2+^ intake^86^ as well as TCR proximal Ca^2+^ concentration.^130^

Although this model provides a simplified overview of TCR activation, the model fails to explain how ITAMs in CD3δ and CD3γ remain protected from tyrosine phosphorylation, as they lack membrane-binding residues,^126,131^ and how a basal level of CD3ζ tyrosine phosphorylation is maintained if they remain to be sequestered in the plasma membrane.^60,117,120,132–134^ Thus, complete sequestering to the plasma membrane might be an unlikely event. Rather, dynamic switching between membrane binding and cytosolic release might happen, and other forces may also be involved.^135^

Constitutive association of ZAP-70 to the CD3ζ might also go against the model of complete sequestering, as ZAP-70 association is mediated through the SH2 domain and phosphotyrosine residues.^132^ Furthermore, constitutively active LCK localized at the cell membrane was detected in up to 40% of resting T cells.^53^ Constitutively LCK activation is probably required for maintaining basal tyrosine phosphorylation of CD3ζ, as T cells with reduced constitutively LCK activation displayed undetectable levels of CD3ζ tyrosine phosphorylation.^53^ But why is TCR signaling not triggered in resting cells if free LCKs are available and apparently are more active than coreceptor-bound LCKs^136^? Perhaps membrane association CD3ζ limits the accessibility to the tyrosine sites in ITAMs, and, if they are accessible in resting cells, CD3ζ orientation allows phosphatases to remove tyrosine phosphorylation.^123^ Therefore, the association of pMHC to the TCR complex that mediates structural changes of the cytosolic part of CD3 is important for TCR activation.

A two-stage kinetics of TCR–pMHC–CD8 interaction has been suggested, where the first TCR binds with the pMHC (within <0.1 s), and then the tri-molecular complex is formed.^137^ The tri-molecular complex was affected by proximal signaling, as pharmacological inhibition of SFKs or CD45 abolished initiation of high-affinity binding.^137^ LCK association to the coreceptor stabilizes coreceptors by preventing endocytosis.^94^ However, how the kinase activity of LCK stabilizes the tri-molecular complex remains to be determined. As an early event, CD8 interacts with CD3ζ, which is independent of pMHC but LCK-dependent, and in the later events, tri-molecular complex is required to maintain the CD8–CD3ζ interaction.^138^ As LCK can be either free or CD8 bound,^138^ CD3ζ might get a chance to meet either free LCK or coreceptor bound LCK before ligand association, probably further explaining CD3ζ tyrosine phosphorylation in resting cells.

A single-molecule analysis suggests that the movement of LCK during TCR activation is not directed but is rather a Brownian movement.^139^ Therefore, it would be challenging for a TCR complex to find LCK instantly unless LCK is already recruited to the complex in resting cells. Free LCKs which are also membrane-bound display higher mobility than coreceptor-associated LCKs, probably due to the difference in molecular size.^136^ This might also explain why free LCKs are recruited in the early TCR complexes.^138^ Besides the ability to move faster, free LCKs display higher catalytic activity as measured by tyrosine phosphorylation (LCK-Y^394^).^136^ However, in any case, coreceptor-bound LCK is required for TCR activation, and the number of coreceptors-bound LCK increases during the maturation process.^140^ Finally, it has been demonstrated that LCKs directly interact with CD3ε in which the interaction is ionic and is mediated through the juxtamembrane basic residue-rich sequence (BRS) of CD3ε and the unique domain (UD) of LCK.^141^

The early steps of the TCR activation process seem to be highly debated. Current models for TCR activation either considered CD3 membrane sequestering and ignored the basal level of CD3ζ or the other way around.^126,127,135^ Perhaps both of the conditions simultaneously occur, and therefore probably CD3ζ holds its states as membrane-embedded and outside the membrane, allowing constitutively active LCK to phosphorylate tyrosine residues on CD3ζ ITAMs (Fig. 2). Then ZAP-70 binds to the phosphotyrosine residues in CD3 ITAMs. Once bound, ZAP-70 is phosphorylated by LCK, which leads to its activation.^142^ This results in the formation of a multi-nucleated signaling complex as further phosphorylation of ZAP-70 allows binding of additional proteins and adaptors, thereby itself behaving as a scaffold.^142,143^ Thus, the activated state of TCR is characterized by phosphorylation of ITAMs, followed by phosphorylation and activation of ZAP-70.

## Distal TCR signaling

**Fig. 3:**
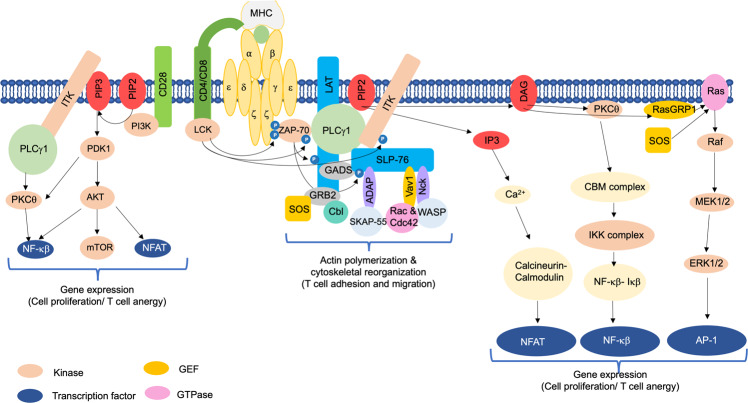
Positive regulation of T cell signaling. The figure depicts the activation of various enzymes and adaptor molecules upon engagement of TCR with the MHC antigenic peptide complex. The phosphorylation events carried out are depicted as small, blue-colored circles. Black lines with arrows indicate activation.

Engagement of TCR with the MHC-antigenic peptide complex of APCs triggers the formation of multi-molecular signalosomes at TCR. This leads to the generation of proximal signaling, followed by the activation of multiple distal signaling cascades, such as Ca^2+^–calcineurin–NFAT, PKCθ–IKK–NFκβ, RASGRP1–RAS–ERK1/2, and TSC1/2–mTOR, with the help of secondary messengers, enzymes, and various adaptor proteins (Fig. 3). These signaling cascades finally bring out the diverse phenotypic effects, as they control many aspects of T cell biology.^1^

### Ca^2+^–calcineurin–NFAT pathway

Phospholipase Cγ1 (PLCγ1) is the main molecule connecting the TCR proximal to distal signaling cascades.^144^ The membrane-bound phosphatidylinositol 4,5-bisphosphate (PIP_2_) gets hydrolyzed by activated PLCγ1 into diacylglycerol (DAG) and inositol-3-phosphate (IP_3_).^145^ Both of these essential secondary messengers initiate a variety of distal signaling cascades important for T cell activation. Membrane-bound DAG can activate PKCθ, RASGRP1, and PDK1-mediated pathways. On the other hand, IP_3_ triggers the activation of a Ca^2+^-dependent calcineurin NFAT pathway.^145–147^

IP_3_ generated from PIP2 binds to the Ca^2+^-permeable ion channel receptors (IP3R) on the endoplasmic reticulum (ER), thereby releasing ER Ca^2+^ stores in the cytoplasm.^148^ It has been found that ERs can sense the intracellular Ca^2+^ levels through the constitutive expression of a transmembrane protein called stromal interaction molecule (STIM). Depletion of intracellular Ca^2+^ levels thus triggers an influx of extracellular Ca^2+^ into T cells from Orai1 type plasma membrane calcium-release activated calcium (CRAC) channel.^149–152^ Increased intracellular Ca^2+^ activates a protein phosphatase, calcineurin, that dephosphorylates the nuclear factor of activated T cells (NFAT), thereby causing its nuclear translocation. Nuclear NFAT forms a complex with AP-1 transcriptional factors (JUN/FOS) derived from the DAG–RAS–MAPK–ERK1/2 pathway. This transcriptional complex is responsible for inducing the expression of various genes, like IL-2 and other effector molecules, that are responsible for T cell activation. In contrast, in the absence of AP-1, NFAT alone activates various genes, like several ubiquitin ligases and diacylglycerol kinase α (DGKα), that are responsible for T cell anergy, a state of T cell unresponsiveness, one of the processes to induce immune tolerance.^153–156^ Thus, two opposite T cell functions—activation and anergy—are controlled by NFAT proteins.^153^

In addition to calcineurin, Ca^2+^ also activates a Ca^2+^/calmodulin-dependent kinase (CaMK) that mediates T cell activation through activation of transcription factors, such as cyclic-AMP-responsive-element-binding protein and myocyte enhancer factor 2.^157^ A missense mutation in Orai1 can lead to impaired Ca^2+^ signaling, which affects nuclear translocation of NFAT, and thereby NFAT-induced cytokines production causes severe combined immunodeficiency (SCID) in humans.^158,159^ Thus, the universal secondary messenger Ca^2+^ regulates several important functions, including proliferation, differentiation, and cytokine production in T cells.^160^

### PKCθ–IKK–NF-κβ pathway

Protein kinase C (PKC) is a family of ten protein serine/threonine kinases that plays numerous roles in physiological and pathological conditions.^161–164^ PKC family proteins are divided into three subfamilies: classical (PKCα, PKCβ1, PKCβ2, and PKCγ), novel (PKCδ, PKCε, PKCη, and PKCθ), and atypical (PKCζ and PKCι). DAG and Ca^2+^ regulate activation of classical PKC isoforms, and novel PKC isoforms are regulated by DAG while atypical PKC isoforms are independent of DAG and Ca^2+^ for activation.^161^ Several PKC isoforms have been implicated in T cell functions.^165^ For example, the novel isoform PKCθ binds to DAG through the PKC conserved region 1 (C1) domain, which is required for its recruitment to the lipid raft after TCR engagement. PKCθ plays major non-redundant roles in T cell activation, even though T cells express several other PKCs.^166,167^

Nuclear factor κβ (NF-κβ) is an evolutionarily conserved transcription factor that plays important roles in regulating genes involved in inflammatory and immune responses, cell growth, survival, and differentiation.^168^ TCR-mediated T cell activation involves both the canonical (classical) and the non-canonical (alternative) NF-κβ pathway. An essential factor required for complete T cell activation via the non-canonical NF-κβ pathway is MAP3K14 (also known as NF-κβ-inducing kinase; NIK).^169^ However, more studies are required regarding this. On the other hand, PKCθ–IKKβ–NF-κβ forms the canonical branch of the NF-κβ pathway and is widely studied.^170^

Once PKCθ is activated following TCR stimulation, it triggers the formation of a tri-molecular complex of adaptor proteins in the cytoplasm called the CBM complex, which is composed of the caspase recruitment domain-containing membrane-associated guanylate kinase protein-1 (CARMA1), B cell lymphoma/leukemia 10 (BCL10), and mucosa-associated lymphoid tissue translocation protein-1 (MALT1).^171^ This is initiated by phosphorylation of CARMA1 by activated PKCθ,^172^ which is required for its oligomerization and association with BCL10.^173^ MALT1 then binds to BCL10, and this association recruits an E3 ubiquitin ligase, called the tumor necrosis factor receptor-associated factor 6 (TRAF6), that polyubiquitinates and degrades IKKγ, or the NF-κβ essential modifier (NEMO), a regulatory protein of the IKK complex.^174,175^ Consequently, the catalytic subunits of Iκβ kinases (IKK), α and β, are no longer inhibited, and they phosphorylate Iκβ, thereby inducing its ubiquitination and degradation. NF-κβ is thus released from its inhibitory Iκβ complex in the cytoplasm, and it translocates into the nucleus to regulate gene expression.^176–178^ This canonical PKCθ–IKKβ–NF-κβ pathway is extremely important for T cell survival, homeostasis, activation, and effector function.^179^ Deregulation of this pathway can cause defective T cell survival and activation, autoimmunity, SCID, and lymphoma.^180–182^

### RASGRP1–RAS–ERK1/2–AP1 pathway

DAG from PIP2 induces the activation of another key molecule, a RAS guanyl nucleotide-releasing protein (RASGRP1), and recruits it to the plasma membrane.^183,184^ RASGRP1 and Son of Sevenless (Sos) are two known guanine nucleotide exchange factors (GEFs) responsible for RAS activation in T cells.^1^ RAS, a small G protein, binds to GTP in the activated state and initiates the RAS-MAPK cascade by activating the serine/threonine kinase Raf1.^183,185^ Raf1, a mitogen-activated protein kinase (MAPK) kinase kinase (MAPKKK), then phosphorylates and activates MAPK kinases (MAPKKs), such as MEK1/2, that further phosphorylate and activate MAPK extracellular signal-regulated kinase-1 & 2 (ERK1/2).^6,185,186^ T cell development, differentiation, and TCR-induced signal strength are all controlled by ERK1/2 signaling.^187–189^ Furthermore, ERKs trigger the phosphorylation and activation of their downstream target Elk, a transcription factor responsible for inducing the expression of c-Fos transcription factor. The VAV1–Rac pathway induces the expression of Jun.^190^ Thus, the formation and activation of a dimeric complex, activator protein-1 (AP-1) composed of Jun/Fos, that plays a critical role in immune response, followed by IL-2 transcription, is sustained by the DAG–RAS pathway.^1,6^ Further, the signal transducer and activator of transcription (STAT3) and LCK get phosphorylated by ERKs.^191,192^

RASGRP1 is extremely important for the development of conventional αβ T cells but not the meager population of γδ T cells.^193–195^ However, it is important for the activation of both types of T cell population as well as for the expression of IL-17.^195^ RASGRP1 deficiency can cause defects in the activation of various signaling pathways, such as RAS–MAPK–ERK1/2, mTOR, and PI3K/AKT.^193,196^ Moreover, abnormal expression of both RASGRP1 and RAS was described in T cells of systemic lupus erythematosus (SLE) patients, thereby implicating the involvement of this pathway in the generation of SLE.^197^

### p38 and JNK pathways

The other MAPKs such as p38 and JNK family proteins play important roles in the proliferation, differentiation, and function of different subsets of T cells.^198–202^ p38 is a family of four highly structurally homolog proteins including p38α (MAPK14), p38β (MAPK11), p38γ (MAPK12), and p38δ (MAPK13).^203^ p38α is widely known as p38 and is one of the most studied isoforms. On the other hand, the JNK family is composed of three members which include JNK1 (MAPK8), JNK2 (MAPK9), and JNK3 (MAPK10).^204^ Three dual-specificity MAP2Ks including MKK3 (MAP2K3, MEK3), MKK4 (MAP2K4, MEK4), and MKK6 (MAP2K6, MEK6) are involved in p38 activation through phosphorylation on the conserved T180-X-Y182 motif in the loop of the substrate recognition site.^203^ Among those three MAP2Ks, MKK3 and MKK6 display higher specificity to p38 while MKK4 also activates JNKs.^203–206^ The conserved T180-X-Y182 motif is required to be phosphorylated on both threonine and tyrosine residues for p38 activation.^207^ In mammalian cells, the classical p38 pathway is regulated by ten different MAP3Ks that allow for the integration of various signaling nodes. However, in T cells, p38 activity is mediated by a non-classical pathway which is downstream of proximal TCR signaling and probably independent of MAPK cascades.^203^ In such a case, activation of TCR proximal signaling results in the phosphorylation of p38 at Y323 residue by ZAP-70, which triggers autophosphorylation on regulatory residues (T180-X-Y182) followed by p38 activation.^208^ Additionally, activated p38 mediated phosphorylation of ZAP-70 on T293 residue may act as a negative feedback loop possibly by limiting excessive TCR signaling.^209^ JNK activation is likely mediated through the activation of PKCθ and CBM complex upon the activation of TCR proximal signaling (reviewed in ref. ^210^). BCL10 oligomers in the CBM complex can recruit TAK1 (MAP3K7), MKK7 (MAP2K7), and JNK2, leading to the activation of JNK.^210,211^

### TSC1/2–mTOR pathway

Upon TCR engagement, both DAG–RASGRP1–RAS–ERK1/2 and PI3K–AKT pathways induce the activation of mTORC1 and mTORC2 (refs. ^196,212^) that differentially regulate the generation of CD4^+^ helper effector T cell types (Th).^213^ mTORC1 down-regulates SOCS5 to promote STAT3 activity, thereby promoting Th17 differentiation.^214^ Resultingly, mice having Rheb or raptor-deficient T cells show defects in Th17 differentiation.^214,215^ On the other hand, mTORC2 phosphorylates AKT at S473 and PKCθ at S660/676 to induce Th1 and Th2 differentiation respectively.^215^ Thus, mice having rictor-deficient T cells show defects in differentiation of IFNγ-producing Th1 and IL-4-producing Th2 effector cells.^214,215^ The activity of mTOR subsequently needs to be tightly controlled, as it regulates T cell activation, differentiation, and function.^216^

As discussed above, the CBM complex plays a crucial role in TCR signaling by recruiting key signaling mediators. Hamilton et al.^217^ demonstrated that CARMA1 and MALT1 in CBM complex, but not BCL10, are required for optimal activation of mTOR in T cells. Furthermore, the CBM complex is involved in TCR-induced glutamine uptake and the activation of mTOR pathways.^218^ Nevertheless, mTOR regulates intracellular metabolic signaling which links to biosynthetic and bioenergetic metabolisms (reviewed in refs. ^219–221^).

## Signaling by the co-stimulatory molecules

TCR engagement leads to activation of proximal and distal signaling pathways. However, productive T cell activation also involves the engagement of additional cell surface receptors, i.e., co-stimulatory molecules like CD28. This is required to avoid anergy, a state of T cell unresponsiveness where T cells become refractory to restimulation by IL-2. If the TCR signals are weak, it results in cell death or anergy. These weak TCR signals are amplified strongly by CD28 engagement, thereby resulting in cell proliferation and differentiation. However, only CD28 engagement results in the expression of a few genes transiently with no biological consequences.^222^

The key event coupling CD28 to several downstream signaling pathways is the recruitment of phosphatidylinositol-3-kinase (PI3K) to the phosphorylated cytoplasmic tail of CD28, which converts PIP2 to PIP3. Once AKT is recruited to PIP3, it acts on several substrates. AKT facilitates prolonged nuclear localization of NFAT, and thus IL-2 transcription, by inactivating GSK-3. IL-2-inducible T cell kinase (ITK) is also associated with PIP3, and this kinase is important for phosphorylation and activation of PLCγ1.^170^ Apart from this, NF-κβ is one of the major signaling pathways regulated by co-stimulation signaling in T cells. AKT associates with CARMA1 and hence facilitates the formation of the CBM complex, which enhances the nuclear translocation and activation of NF-κβ. However, AKT is non-essential for NF-κβ signaling in T cells.^223^ The most important mediator of the NF-κβ signaling pathway is phosphoinositide-dependent kinase-1 (PDK1), whose recruitment and phosphorylation enable its efficient binding to both CARMA1 and PKCθ, thus inducing NF-κβ activation.^224,225^ Indeed, activation of NF-κβ and PKCθ was found abrogated upon deletion of PDK1 in T cells.^224^ VAV1 is a GEF for small GTPases, such as Rac1, Rac2, and Rhog, where it plays a crucial role in strongly amplifying CD28-mediated activation of NFAT and NF-κβ signaling pathways.^226,227^ Thus, signaling by co-stimulatory molecules quantifies the signals that are already activated by TCR ligation, thereby strongly sustaining T cell activation. These include the PI3K–AKT–mTOR, NFAT, NF-κβ, and MAPK pathways (Fig. 3). While PI3K signaling is primarily mediated by CD28, initial activation of PI3K results in upregulation of phosphoinositide-3-kinase adaptor protein-1 (PIK3AP1, also known as BCAP).^228^ PIK3AP1 potentiates PI3K signaling in response to CD3 engagement in CD8+ T cells.^228^

## Positive regulators of T cell signaling

Two of the most important adaptors that are phosphorylated by activated ZAP-70 and play a critical role in positively regulating TCR signaling are a transmembrane adaptor protein, linker for activation of T cells (LAT), and a cytosolically localized SH2 domain-containing leukocyte phosphoprotein of 76 kDa (SLP-76)^229,230^ (Fig. 3). These adaptor proteins form the backbone of the proximal signaling complex (proximal signalosome) that recruits various other effector proteins,^231,232^ along with phospholipase Cγ1 (PLCγ1), which links the proximal with several distal signaling pathways upon TCR engagement.^144^ This results in a stable and dynamic zone of contact between APCs and T cells, designated as the immunological synapse (IS).

Lipid rafts are microdomains located within the plasma membrane that are enriched with cholesterol, glycosphingolipids, and sphingomyelin, and these rafts accumulate at the IS. They are also known as glycolipid-enriched microdomains (GEMs), detergent-resistant membranes (DRMs), or detergent-insoluble glycolipid-enriched membranes (DIGs). Key components of the TCR signaling pathway, such as LCK, LAT, RAS, CD4, and FYN, along with some others, are located within the lipid rafts.^6^ Post-translational modification of lipids in these molecules is very important for their localization in the lipid rafts. RAS is both palmitoylated and farnesylated, whereas most of the SRC PTKs, including the T cell-specific LCK, undergoes myristoylation and palmitoylation, necessary for its localization in the lipid rafts and subsequent targeting and phosphorylation of the CD3 ζ chain.^233–235^ TCR engagement and activation result in its rapid association with the lipid rafts, and this localization is important for the early tyrosine phosphorylation events of the TCR subunits by the SRC family PTKs.^236,237^ This can be achieved by the PTK LCK that is present in the rafts, where its SH2 domain binds to the phosphorylated tyrosine residues in activated ZAP-70, thereby bringing TCRs bearing activated ZAP-70 to the lipid rafts.^238,239^

The GM-CSF/IL3/IL5 common β-chain-associated protein (CBAP) is involved in the regulation of TCR downstream signaling, as CBAP-deficient cells display reduced phosphorylation of PLCγ1, LAT, JNK1/2, and ZAP-70,^240^ suggesting that it may play a role in both proximal and distal signaling. Besides its role in normal physiology, CBAP plays an important role in T cell acute lymphoblastic leukemia (T-ALL) pathology. CBAP was found to be highly expressed in T-ALL, and its expression enhanced T-ALL cell growth.^241^ Similar to TCR signaling, loss of CBAP decreased ERK1/2, S6K, RSK, and TSC2 phosphorylation and thereby decreased aerobic glycolysis and energy metabolism.^241^

### LAT

The first adaptor essential for the successful transmission of TCR signals is the LAT.^13^ It is a transmembrane protein of 36–38 kDa^242^ consisting of a tyrosine-rich cytoplasmic tail and a short extracellular region.^229^ It requires palmitoylation on its two cysteine residues (C26 and C29) for localization to the lipid rafts. Mutation of C26 fully inhibited the localization of LAT to the lipid rafts, whereas C29 mutation had a partial effect. Moreover, no tyrosine phosphorylation of LAT was detected when C26 was mutated, indicating that raft localization of LAT is vital for its phosphorylation.^243^ Several molecules have been proposed that link TCR and LAT by binding to both of them. PLCγ1 binds to phosphorylated tyrosine residue 132 of LAT^244^ via its N-terminal SH2 domain and to phosphorylated tyrosine residues on activated ZAP-70 via its C-terminal SH2 domain.^245^ An Abl-SH3 interacting protein, 3BP2 has also been found to interact with both LAT and ZAP-70 via its SH2 domain, probably in a multimeric form.^246^ Another important molecule found to link TCR and LAT is a small adaptor protein, Shb, that binds to the phosphorylated tyrosine residues on the CD3 ζ chain via its SH2 domain and also binds phosphorylated LAT via its non-SH2 phosphotyrosine binding domain.^247^ TCR engagement results in rapid phosphorylation of LAT on its tyrosine residues by ZAP-70.^248^ Thus, LAT phosphorylation and distal signaling events were inhibited when mutant Shb was expressed with a defective SH2 domain.^247^ Once phosphorylated, LAT then binds to several proteins, such as enzymes and adaptor molecules, via diverse binding sites as discussed below, therein bringing them to the plasma membrane.

Overexpression of LAT did not augment TCR-mediated downstream signaling pathways.^249^ Moreover, LAT-deficient Jurkat cells also displayed TCR-induced receptor phosphorylation and ZAP-70 activation but were found to be defective in all steps distal from this. There was no activation of PLCγ1 with reduction of Ca^2+^ mobilization, ERK activation, NFAT activation, and reduced IL-2 gene transcription.^249,250^ Reconstitution with LAT restored these defects. Moreover, LAT-deficient mice blocked thymic differentiation at the pre-TCR stage, thereby showing no T cells in the lymph nodes and spleen.^250^ LAT, then, is important for TCR-mediated signaling and intra-thymic development of T cells.

### Growth factor receptor-bound protein 2 (GRB2) and GRB2-related adaptor downstream of Shc (GADS)

GRB2 and GADS are cytosolic adaptor proteins, with the former expressed ubiquitously and the latter expressed only in the hematopoietic cells, playing an important role in hematopoietic growth factor receptors signaling.^251,252^ Activated/phosphorylated LAT binds to both the GRB2 family proteins via their SH2 domains, thereby translocating them to the plasma membrane, along with their SH3 domain-associated proteins.^6^ GRB2 is constitutively associated with Sos, a dual-specific GEF for small GTPases such as RAS and Rho. Upon TCR activation, Grb2-Sos complex associates with LAT, leading to activation of RAS. However, Grb2 seems insufficient for RAS activation in T cells, as LAT mutants failed to induce complete RAS activation.^183,253^ An additional small linker molecule, Shc, was found to mediate the association of Sos with GRB2 in T cells.^254^ An E3 ubiquitin ligase, CBL (discussed later), is another GRB2 associated protein that binds to phosphorylated LAT in T cells.^255^ Upon TCR stimulation, another member of the GRB2 family, GADS not only binds to phosphorylated LAT but also specifically binds to a critical adaptor molecule of T cells, SLP-76,^256^ thereby associating LAT with SLP-76. GADS has also been found to associate with a serine/threonine kinase, hematopoietic progenitor kinase-1 (HPK1), involved in JNK pathway activation.^256,257^ T cell development was found impaired, with specific defects in both positive and negative selection of thymocytes, in GADS-deficient mice.^258^

The three distal tyrosine residues of LAT (171, 191, and 226) are involved in binding to Grb2, whereas Tyr 171 and 191 are involved in binding to GADS.^244^ Mutation in any one of the tyrosine residues did not affect either Grb2 or GADS binding, whereas loss of both 171 and 191 decreased GRB2 binding, and mutation of both these residues completely abolished GADS binding. The binding of GRB2 was only abolished when all three tyrosine residues were mutated. Since these tyrosine sites might directly interact with PLCγ1 through its C-terminal SH2 domain or indirectly via GADS–SLP-76–PLCγ1 interaction, mutations of these tyrosine residues also impacted PLCγ1 binding due to loss of SLP-76 binding, with PLCγ1 activation completely inhibited, calcium flux partially inhibited, and PLCγ1-LAT association being undetected.^244^

### SH2 domain-containing leukocyte phosphoprotein of 76 kDa (SLP-76)

SLP-76 is another crucial multidomain adaptor protein of 76 kDa, localized in the cytoplasm^13,230^ and expressed only in cells of the hematopoietic system, such as thymocytes, mature T cells, natural killer cells, megakaryocytes, and macrophages but not B cells.^259^ It plays a very important role by linking LAT, activating PLCγ1, and other downstream signaling pathways.^13^ The proline-rich region of SLP-76 binds to the SH3 domain of PLCγ1,^260^ leading to the formation of the LAT–GADS–SLP76–PLCγ1 complex. Thus, the two complexes described above, LAT-GADS-SLP-76 and LAT-PLCγ1, interact with each other via binding of SLP-76 to PLCγ1.^261^ SLP-76-deficient Jurkat cells subsequently displayed severe impairment of PLCγ1 phosphorylation, resulting in decreased calcium flux and IL-2 production, following TCR engagement.^262^ Overexpression of SLP-76 in Jurkat cells increased TCR-mediated NFAT activity and IL-2 transcription as well as ERK activation, but calcium flux remained unchanged.^263,264^ Moreover, SLP-76-deficient mice showed an intra-thymic block at an early developmental stage of T cells (double negative stage), thereby failing to generate normal, peripheral T cells, displaying the same phenotype as the LAT-deficient mice.^265,266^ Such findings demonstrate that SLP-76, like LAT, is important for TCR-mediated signaling and intra-thymic development of T cells.

Upon TCR stimulation, SLP-76 gets phosphorylated by ZAP-70 at its multiple tyrosine residues, which serve as binding sites for various SH2 domain-containing proteins. These include proteins involved in cytoskeletal rearrangements, such as VAV1, non-catalytic tyrosine kinase (NCK), and the PTK ITK (described below).^267–269^ On the other hand, the SH2 domain of SLP-76 associates with the phosphorylated tyrosine residues of a 130 kDa multidomain adaptor protein, named SLP-76-associated phosphoprotein (SLAP)/FYN-binding protein (FYB)/Adhesion and degranulation promoting adaptor protein (ADAP).^270,271^ SLP-76 thus regulates cytoskeletal changes in activated T cells by coordinated and precise loading of the effector molecules VAV, NCK, and ADAP into the complex, vital for the stability of the complex and its optimal activation.^1^

### Connecting link PLCγ1

Since both LAT and SLP-76 form the backbone of the proximal signaling complex, deficiency of both the adaptors in Jurkat cells and mouse models showed diminished activation of RAS signaling due to impairment in formation of the proximal signalosome.^265,272^ As the connecting link between proximal and distal signaling pathways, PLCγ1 is the central signaling molecule in T cells, and phosphorylation on its multiple tyrosine residues is required for its full activation.^273^ This is mediated by the TEC PTK family members, such as IL-2 inducible T cell kinase (ITK) and resting lymphocyte kinase (RLK)/TXK. Deletion of both ITK and RLK exhibited complete loss of PLCγ1 activity along with defects in calcium flux following TCR engagement.^274–277^ In contrast, overexpression of RLK enhanced PLCγ1 phosphorylation and calcium flux.^278^ Plasma membrane localization of ITK and RLK/TXK is possible via its N-terminal pleckstrin homology (PH) domain and palmitoylation respectively. ITK interacts with many different molecules via its various binding domains. The Tec homology (TH) region of ITK is a proline-rich region that interacts with the SH3 domain of Grb2. The SH3 domain of ITK, in turn, interacts with the proline-rich regions of PLCγ1.^260^ LAT interactions with ITK have also been reported; nevertheless, the exact mechanisms are still unknown.^279^ Moreover, the SH2 domain of ITK interacts with tyrosine-phosphorylated SLP-76.^268,269^ Thus, ITKs can interact with the LAT-associated molecules via multiple mechanisms. ITKs in turn are activated by both SLP-76 and LCK.^280,281^ When ITK associates with SLP-76, it is present in close proximity to its substrate PLCγ1, and direct phosphorylation of ITK at Y511 by LCK promotes its activation.^269,282^ Moreover, apart from ITK, the association of PLCγ1 with LAT, GADS, and SLP-76 is also required for its optimal activation.^253^ In response to TCR engagement, PLCγ1 activation is thus regulated by the signaling complex (signalosome) composed of LAT, GADS, SLP-76, PLCγ1, and ITK.^6^

### Signaling lymphocyte activation molecule (SLAM)-associated protein (SAP)

Signals from both the TCR-CD3 complex and co-stimulatory receptors, such as CD28, CD2, and the CD150/SLAM (signaling lymphocyte activation molecule) family, are required for full activation of T cells.^283^ SAP is a small cytoplasmic protein of 128 amino acids^284^ that associates via its SH2 domain with the immunoreceptor tyrosine-based switch motifs (ITSMs) present in the cytoplasmic tail of the SLAM family of receptors. Once bound to a specific ITSM, it may prevent binding of the SH2 domain-containing protein tyrosine phosphatase 2 (SHP-2) and thereby compete with it. On the other hand, it may favor the recruitment of SH2 domain-containing inositol phosphatase (SHIP), causing the switch between these two signaling pathways.^285,286^ The SLAM family consists of a number of transmembrane costimulatory receptors, such as CD150/SLAM, CD244/2B4, CD84, CD229/Ly-9, CD319/CRACC, and NTB-A.^287^ Thus, SAP can bind via its SH2 domain to the ITSMs of various SLAM families of receptors, and this interaction plays a crucial role in mediating the costimulatory signals necessary for T cell activation.^283^ Moreover, SAP also exerts its adaptor role by binding to various SH3 domain-containing proteins, such as FYN, PKCθ, βPix, and NCK1,^288–291^ thus recruiting them to the SLAM family of transmembrane receptors.^292^

Along with SLAM, SAP has also been found to directly associate with the first ITAM (Y72-Y83) of the CD3ζ chain in various T cell lines and peripheral blood lymphocytes. Knockdown of SAP resulted in a decrease of several canonical T cell signaling pathways, such as AKT and ERK; reduced the recruitment of PLCγ1, SLP76, and Grb2 to the phosphotyrosine containing complex; and also reduced IL-2 and IL-4 mRNA induction. Through its direct association with the CD3ζ chain, SAP was found to play a central role in T cell activation.^292^ Indeed, mutations or deletions of the SH2D1A gene encoding SAP resulted in X-linked lymphoproliferative syndrome-1 (XLP1), which is characterized by immunodeficiency due to a specific defect in T cells (apoptosis resistance and impaired interaction with B cell), reduced cytotoxicity of natural killer cells, a decrease in B cell functions, and defective NKT cell development.^293,294^

SAP and NTB-A (SLAMF6) are essential proteins that potentiate the strength of proximal TCR signals required for restimulation-induced cell death (RICD).^295^ RICD is an important consequence of repeated TCR signaling essential for TCR-induced apoptosis in thymocytes, mature T cells, T cell malignancies, and T cell therapies (reviewed in refs. ^296–298^). Therefore, T cells with impaired SAP function display resistance to RICD, which likely explains severe CD8+ T cell lymphoproliferation in XLP1 patients.^295^

## Negative regulators of T cell signaling

**Fig. 4:**
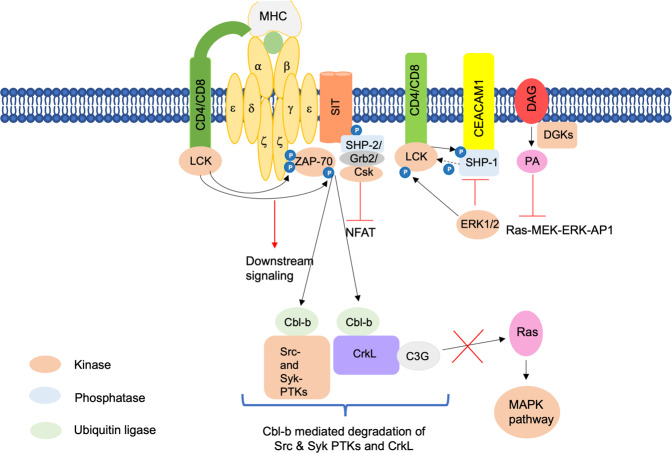
Negative regulation of T cell signaling. The figure depicts various adaptors and enzymes, like kinases and phosphatases, involved in negatively regulating TCR signaling. The phosphorylation events carried out are depicted as small, blue-colored circles. Black lines with arrows indicate activation. Dotted black lines with arrows indicate dephosphorylation events.

Inappropriate activation of T cells is prevented by the termination of TCR signals, and this is mediated by certain proteins that negatively regulate TCR signaling (Fig. 4).

### Adaptors serving as negative regulators

#### Phosphoprotein associated with glycosphingolipid-enriched microdomains (PAG)/CSK-binding protein (CBP)

An important transmembrane adaptor protein negatively regulating TCR signaling is PAG/CBP which is found in the lipid rafts.^299,300^ In the absence of TCR engagement or in resting T cells, PAG is constitutively tyrosine phosphorylated in its cytoplasmic tail. This serves as a docking site for the SH2 domain of the major negative regulator of SRC kinases, the tyrosine kinase c-terminal SRC kinase (CSK), thereby localizing to the rafts and activating.^301–303^ Once activated, CSK phosphorylates LCK at the C-terminal Y505 residue, which leads to its kinase domain inactivation as it causes LCK to bind to its internal SH2 domain.^49,69,301–303^ Thus, CSK gets activated upon binding to PAG in the lipid rafts, and it inhibits the activity of SRC family kinases. However, upon TCR activation, tyrosine phosphatase CD45 transiently dephosphorylates PAG. This results in the dissociation of CSK from the glycosphingolipid-enriched microdomains (GEMs), relieving the inhibition of SRC kinases for signal transmission.^304^ Moreover, the inhibitory Y505 residue of LCK also gets dephosphorylated by CD45 tyrosine phosphatase, which, furthermore, slightly dephosphorylates positive regulatory autophosphorylation at Y394.^53,305^ Thus, the PAG-CSK complex maintains T cell quiescence by transmitting negative regulatory signals.^13^

#### SH2 or SHP-2-interacting transmembrane adaptor protein (SIT)

Another transmembrane adaptor protein negatively regulating TCR signaling is SIT expressed in lymphocytes.^306–308^ It associates with the TCR complex as a disulfide-linked homodimer.^306^ The cytoplasmic tail of SIT contains immunoreceptor tyrosine-based inhibition motifs (ITIMs) that, upon tyrosine phosphorylation, associate with SHP-2. SIT mediates its negative regulation of TCR signaling through the inhibition of NFAT activity. That, however, remains unaffected after mutation of the tyrosine residue within the ITIM motif, which completely abrogates binding to SHP-2. Thus, SIT-SHP-2 interaction seems unimportant for SI-mediated negative regulation of T cell signaling.^306^ GRB2 was also found to be associated with SIT via two consensus YxN motifs whose mutations abrogated the binding. This also had no effect on the inhibitory function of SIT.^308^ Moreover, the effector molecule that might mediate the negative regulatory function of SIT was found to be CSK via co-precipitation experiments.^308^ However, the precise mechanism of SIT-mediated negative regulation of TCR signaling needs to be elucidated,^13^ as its role in lymphocyte function seems to be more complex.^10^

### Enzymes serving as negative regulators

Enzymes such as phosphatases, kinases, and ligases also play important roles in negatively regulating TCR signaling.

#### Phosphatases

Apart from CD45 and SHP-2 already mentioned above, there are other tyrosine phosphatases that mediate negative regulation. Adhesion molecules called carcinoembryonic antigen-related cell adhesion molecule-1 (CEACAM1) are expressed at later time points of TCR stimulation.^309^ The SH2 domain-containing protein tyrosine phosphatase 1 (SHP-1) is recruited to the phosphorylated ITIMs of CEACAM1, and it dephosphorylates LCK at Y394, inactivating it^57,309,310^ and thus terminating TCR signaling. The binding of TCR to antagonists or weak antigens induces LCK-mediated phosphorylation of SHP-1 at Y564, thereby activating it, which, in turn, dephosphorylates and inactivates LCK.^309,310^ Moreover, SHP-1 binding to LCK is prevented by ERK1/2-mediated phosphorylation of LCK at S59, sustaining TCR signaling. SHP-1 activity is thus indirectly regulated by ERK1/2.^311^ Additional phosphatases that negatively regulate TCR signaling include PTEN, which dephosphorylates PIP3, and dual-specificity phosphatases, which dephosphorylate MAPKs.^312,313^

#### Diacylglycerol kinases

Subcellular levels of DAG are regulated by lipid kinases, DGKs that phosphorylate DAG to produce phosphatidic acid (PA).^314,315^ Consequently, the increase in DGK activity attenuates RAS–MEK–ERK–AP1 signaling induced by TCR-mediated DAG activation.^316,317^ Ten DGK isoforms are expressed in mammals,^315^ with DGK α and ζ being expressed at high levels in T cells.^318^ Both isoforms negatively regulate the DAG–RASGRP1–RAS–ERK1/2 pathway and thus inhibit activation of mTORC1 and mTORC2 complexes.^196^ Indeed, the genetic ablation of these isoforms resulted in increased activation of the RAS–MEK–ERK–AP1 pathway, mTOR signaling, and PKCθ–NF-κβ pathway. This led to the loss of T cell anergy and increased T cell hyperactivation.^145,155,156,319,320^ Both DGK α and ζ perform redundant roles in T cells, as their deficiency resulted in severe T cell developmental blockade at the DP stage, which was partially restored with the phosphatidic acid treatment.^321^ DGK activity can be regulated by SAP. Overexpression of SAP reduces DGKα activity which was shown to be dependent on the SH3-binding ability of SAP in T cells, suggesting that SAP acts as a negative regulator of DGKα.^322^ As SAP-deficient XLP1 displays resistance to RICD, pharmacological inhibition of DGKα in SAP-deficient cells can restore RICD, indicating that XLP1 patients will likely benefit from DGKα-targeted therapy.^295,323,324^

#### E3 ubiquitin ligases

E3 ubiquitin ligases are enzymes that ubiquitinate different proteins and target them for proteasomal- or lysosomal-mediated degradation.^325^ Some of these ligases regulate T cell tolerance, and T cells can become autoreactive upon their deletion or mutation, leading to autoimmunity.^326^ TRAF6, as mentioned before, is one such E3 ubiquitin ligase that plays an important role in the activation of the NF-κβ signaling pathway.^174,175^ Itch is another ubiquitin ligase that not only targets PLCγ1 and PKCθ^327,328^ but also Jun, thereby causing diminished activation of AP-1.^329^ Itch thus regulates T cell anergy by degrading certain components of TCR signaling.^330^ Itch-deficient mouse models are therefore prone to autoimmune and pro-inflammatory phenotypes.^1^

A well-studied E3 ligase that marks various proteins for ubiquitin-mediated degradation is Casitas B cell lymphoma (CBLB). This enzyme, along with c-CBL, another member of the CBL family, negatively regulates TCR signaling.^331^ In activated T cells, the CD3ζ chain gets ubiquitinated by CBLB at its multiple lysine residues and induces degradation of surface TCRs.^332–335^ Some other targets for CBL-mediated ubiquitination and protein degradation include members of the proximal signaling complex, SRC- and Syk-family PTKs,^336,337^ the regulatory p85 subunit of PI3K,^338^ and the adaptor molecule VAV1.^337^ All these events result in the attenuation of TCR signaling. In another mode of action, T cell activation leads to dissociation of CBLB from Grb2, and it then binds to CRKL, an adaptor molecule required for T cell adhesion and migration. CRKL is in turn constitutively associated with C3G, a GEF for the small GTPases such as RAP1, RAP2, and R-RAS.^339–345^ The CRKL–C3G–RAP1 signaling pathway increases the affinity of β1-integrins to the extracellular matrix (ECM), regulating and mediating the adherence of the hematopoietic cells to ECM and stromal cells.^346,347^ Cell proliferation, cytoskeletal reorganization, and cell-to-cell contact are some of the most critical biological effects of the CRKL–C3G–Rap1 signaling pathway.^341,346,348^ The binding of CBL to CRKL results in the ubiquitination of CRKL, thus disrupting the CRKL–C3G–Rap1 signaling. On the other hand, an increase in CRKL–C3G–RAP1 signaling, along with clustering of the integrin, lymphocyte function-associated antigen 1 (LFA-1), was observed in response to TCR engagement upon knockdown of CBLB.^349,350^ CBLB thus serves as the negative regulator of CRKL–C3G–RAP1-mediated signaling events that promote T lymphocyte adhesion, migration, and homing.^351^ Both LCK and FYN seem to be involved in TCR downregulation, as pharmacological inhibition of LCK and FYN led to stabilization of the TCR complex.^352^

## Adaptor proteins in cytoskeletal reorganization

Early events leading to T cell activation also involve cytoskeletal changes required for lymphocyte migration and mediating cell-to-cell adhesion.^353^ Interaction of T cells and APCs results in T cell activation, which involves supramolecular rearrangement of a number of receptors at the contact zone, thus forming a synapse. Initially, the integrin receptors of T cells and integrin receptor ligands of APCs are present in the center surrounded by a ring of MHC-peptide complexes. However, this pattern completely reverses within a few minutes, and MHC-peptide complexes form the central region known as the central supramolecular activation cluster (cSMAC), surrounded by integrin receptors in the periphery, forming the peripheral supramolecular activation cluster (pSMAC).^354–356^ These structures are stable for a few hours where specific molecules can be detected. For example, PKCθ has been detected in cSMAC,^354^ whereas CD45 is initially excluded from cSMAC only to migrate back to it later.^357^ These molecular rearrangements are partly regulated by the cytoskeleton,^354,355^ where a ring of polymerized actin accumulates in T cell-APC conjugates or at the interface between T cells stimulated with anti-TCR antibodies.^358^ LAT and all its binding partners, such as PLCγ1, GADS, and GRB2, are essential for efficient actin polymerization, as the absence of LAT or mutation in binding sites of either of the components inhibits polymerization of actin.^358^ SLP-76 is one of the molecular adaptors present in a complex with LAT in activated T cells. It binds to several different adaptors involved in regulating the cytoskeleton. Two such adaptors, VAV and NCK, bind to the amino-terminal phosphotyrosine residues of SLP-76 (refs. ^359,360^) to promote cytoskeletal reorganization, whereas ADAP binds to the carboxyl-terminal phosphotyrosine residues of SLP-76 to promote integrin signaling^361^ (Fig. 1).

### VAV

VAV structurally is a multidomain adaptor protein and functionally a GEF for the activation of Rac and Cdc42, members of the Rho/Rac family of small GTPases.^362^ Defects in IL-2 production and partial blocks in calcium mobilization were seen upon targeted disruption of VAV. VAV-deficient T cells also showed defects in cytoskeletal function,^363,364^ along with impaired SMAC formation.^365^ LAT-deficient T cells would thus fail in the recruitment of VAV via SLP-76, thereby decreasing the amount of activated Rac and Cdc42. This could result in inadequate activation of phosphatidylinositol 4-phosphate 5-kinase, an enzyme responsible for generating PIP2, a substrate of PLCγ1.^366^ Moreover, there could be inadequate activation of Wiskott–Aldrich syndrome protein (WASP) due to insufficient Rac activation.^6^

### Wiskott–Aldrich syndrome protein

WASP, as the name suggests, was initially identified as a defective protein from Wiskott-Aldrich syndrome patients.^367^ Decreased IL-2 production, calcium flux, and defective actin polymerization were seen in VAV−/− animals, and a similar phenotype was observed in T cells from WASP patients and murine cells from WAS−/− animals.^368,369^ This could be explained by the fact that WASP normally exists in an autoinhibited state in resting T cells, where the GTPase-binding domain interacts with the C-terminus that contains the Arp2/3 complex responsible for actin polymerization. The effectors of VAV, RAC, and CDC42, along with PIP2, upon activation, associate with WASP, thereby synergistically activating it and stimulating actin polymerization through the Arp2/3 complex.^370,371^ WASP is also associated with the NCK, which in turn binds SLP-76.^372^ Thus, SLP-76 might act as a scaffold by binding to both the NCK and VAV, thereby bringing WASP, RAC, and CDC42-GTP into close proximity so that they can interact with each other.^373^

### NCK and T cell-specific adaptor protein (TSAd)

NCK is another adaptor protein known to regulate the actin cytoskeleton that constitutively interacts with VAV1.^374^ Both NCK and VAV1 further interact with SLP-76 upon TCR engagement, thereby forming a complex that associates with components of the TCR-CD3 complex, leading to reorganization of actin at the T cell-APC interface.^360,373,375^ A T cell-specific adaptor protein, TSAd, mediates the association of NCK with LCK and SLP-76 in T cells, thus controlling actin polymerization events in activated T cells. Both NCK and TSAd were found to co-localize in Jurkat cells, where NCK, via its SH2 and SH3 domains, interacts with pTyr280 and pTyr305 and the proline-rich region (PRR) of TSAd respectively. Further, increased polymerization of actin was observed in Jurkat cells expressing TSAd, and this was due to the presence of TSAd exon 7, which encodes interaction sites for both NCK and LCK.^376^

Moreover, many proteins tend to associate with NCK, as more than 60 binding partners have been identified.^377,378^ Thus, TSAd may influence the actin cytoskeleton by bringing LCK in the vicinity of different NCK binding partners.^376^ Moreover, CXCL-12-induced migration and cytoskeletal rearrangements in T cells are regulated via TSAd by promoting LCK-mediated tyrosine phosphorylation of ITK.^379^ ITK not only binds to TSAd^281,379^ but also SLP-76,^375^ thereby forming a multiprotein complex of NCK, TSAd, LCK, SLP-76, and ITK that may interact with each other and several other molecules in a cooperative manner.^376^ NCK thus plays a vital role in the regulation of the actin cytoskeleton, IS formation after TCR engagement, and cell proliferation and migration.^377,380,381^

### Adhesion and degranulation promoting adaptor protein (ADAP)

Another component of the actin polymerization machinery in T cells is the SLP-76-associated phosphoprotein of 130 kDa (SLAP-130)/FYN-binding protein (FYB)/adhesion and degranulation promoting adaptor protein (ADAP).^6^ It is a multidomain adaptor protein that, upon TCR engagement, gets phosphorylated by SRC family kinases, such as FYN, enabling its binding to the SH2 domains of SLP-76 and FYN.^382,383^ Peripheral T cells deficient in ADAP demonstrated defects in cell proliferation, cytokine production, and clustering of the integrin LFA-1 upon TCR stimulation,^270,271^ whereas TCR-driven IL-2 transcription was increased upon co-transfection of ADAP with SLP-76 and FYN.^383,384^ Integrin clustering with the help of ADAP facilitates T cell migration in response to stromal cell-derived factor 1 alpha (SDF1α)^385^ and enhances T cell-APC conjugate formation.^386^ ADAP thus couples TCR-mediated actin cytoskeletal changes to integrin activation.^13^ ADAP associates with proteins of the Ena (enabled)/VASP (vasodilator-stimulated phosphoprotein) family, important for the regulation of actin dynamics and T cell polarization, thereby regulating cell adhesion mediated by integrins.^387^ ADAP also interacts with a multiprotein complex composed of WASP, Arp2/3, VAV, NCK, and SLP-76. TCR-mediated actin rearrangement was inhibited when the binding between the WASP and Arp2/3 complex or ADAP and Ena/VASP proteins was hindered, suggesting that TCR signaling is linked to cytoskeletal remodeling by these interactions.^388^

### SRC kinase-associated phosphoprotein of 55 kDa (SKAP-55)

SKAP-55 or Scap1 is a T cell-specific adaptor protein that is constitutively associated with ADAP.^389,390^ It enhances cellular adhesion by not only promoting the clustering of LFA-1 but also enhancing its binding to intercellular adhesion molecule-1 (ICAM-1) and fibronectin. SKAP-55 also increases T cell/APC conjugate formation, thereby inducing its translocation to the lipid rafts.^386^ This brings it into close proximity with the SRC kinase FYN that phosphorylates SKAP-55.^391^ Both ADAP and SKAP-55 might control the formation of SMACs since they enhance LFA-1-mediated adhesion during T cell/APC interactions, which are important for SMAC formation.^13^

### Mammalian CT10 (chicken tumor virus number 10) regulator of kinase (CRK)

The CRK adaptor proteins are ubiquitously expressed and regulate proliferation, differentiation, adhesion, migration, and apoptosis of immune cells by integrating signals from various effector molecules, such as ECM, growth factors, pathogens, and apoptotic cells.^392–396^ There are three members belonging to the family of CRK adaptor proteins, CRKI, CRKII, and CRK-like (CRKL), that mediate various protein interactions through their SH2 and SH3 domains. Transient interactions with STAT5, ZAP-70, CBL, and CASL (CRK-associated substrate lymphocyte type) are mediated by the CRK SH2 domains, thus activating the lymphocytes. Cytokines secreted upon TCR activation can induce STAT5 tyrosine phosphorylation, possibly through Janus kinase 3 (JAK3), which was shown to be required for T cell proliferation.^397^ Furthermore, the STAT5 function is required for amino acid biosynthesis.^398^ Cell adhesion of lymphocytes, their extravasation, and recruitment to the sites of inflammation are mediated by the constitutive association of CRK with C3G via their SH3 domains. A detailed function of CRKL-C3G is mentioned in the ubiquitin ligase section of this paper, while the reader is also referred to the comprehensive review on the role of CRK adaptor proteins in T cell adhesion and migration.^399^

## TCR signaling dysregulation in diseases

Dysregulation of TCR signaling can lead to the generation of various diseases, given its importance in executing different functions of T cell biology. Thus, defects in TCR signaling can lead to immune deficiency. On the other hand, its hyperactivation can lead to autoimmune diseases. TCR signal transduction is thus tightly regulated via multiple mechanisms by various enzymes and non-enzymatic proteins that serve as scaffolds for efficient signal transmission.^183^ Mutations in any of these mediators can contribute to the dysregulation of TCR signaling, leading to various disorders.

Both immune deficiency and autoimmunity have been observed when tyrosine phosphatase CD45 is misexpressed. SCID which is characterized by the absence or defective function of T cells, displays deficiency of CD45 expression,^400,401^ whereas multiple sclerosis (MS) is an end result of certain CD45 polymorphisms.^402^ SCID was also generated due to mutations in genes coding for CD3 δ, ε, and ζ chains.^403^ Both mice and humans showed immunodeficiency due to defective expression of LCK.^404,405^ Furthermore, a rare form of SCID was observed in humans with functionally impaired CD4^+^ T cells and the absence of CD8^+^ T cells due to deficiency or mutation of ZAP-70.^406,407^ In contrast, the development of T cells at the CD4^+^ CD8^+^ double-positive stage in mice was blocked due to deficiency of ZAP-70, thereby having a complete absence of single positive CD4^+^ and CD8^+^ T cells.^1^ On the other hand, autoimmune disorders can be caused by an abnormal thymic selection of T cells or their uncontrolled proliferation due to dysregulated TCR signaling. Rheumatoid arthritis (RA) and SLE have been found to be associated with reduced expression of CD3ζ.^408,409^ Similar to human RA, autoimmune arthritis has been observed in mice having spontaneous mutations in the SH3 domain of ZAP-70.^410^ Non-T cell activation linker (NTAL) is a transmembrane adaptor molecule that enhances methylprednisolone and TCR-induced apoptosis in T-ALL through increased ERK phosphorylation.^411^ NTAL thus serves as a tumor suppressor in T-ALL, where its high mRNA expression correlates with a good response to prednisone and vice versa.^412^ Accordingly, NTAL^−^^/−^ mice displayed activated T cells characteristic of an autoimmune syndrome.^411^

Peripheral T cell lymphomas (PTCL) and T cell acute lymphoblastic leukemias or lymphomas (T-ALL) both constitute different groups of T cell malignancies; PTCL arises from post-thymic mature T cells whereas T-ALL arises from thymic immature T cells blocked at various stages of development.^413^ Multiple molecular aberrations have been described in genes involved in TCR signaling in PTCL,^413^ with 84% of Sezary syndrome samples^414^ and 90% of adult T cell leukemia/lymphoma samples^415^ showing mutations in TCR signaling components. Upregulation of the LAT adaptor along with frequent activating mutations (gain-of-function alterations) in the adaptor CARD11 and PLCγ1 have been observed in most cases of Sezary syndrome cutaneous T cell lymphoma,^414^ contributing to cell survival and proliferation and disease progression. Thus, the TCR signaling in this context is oncogenic. In contrast, translocations involving TCR genes have been identified in T-ALL^416^ with no recurrent mutations in any of the TCR signaling components.^413^ In a landmark study, Trinquand et al. in 2016^417^ identified that TCR engagement with an MHC-restricted TCR-specific antigen or via CD3 stimulation with anti-CD3 antibody OKT3 made TCR-positive T-ALL cells undergo apoptosis in a similar transcriptional program as the thymic negative selection. However, leukemia recurrence was observed in TCR-positive T-ALL xenografts due to the presence and selection of TCR-negative subclones as a mechanism of tumor relapse from OKT3-mediated therapy. Nevertheless, it is quite encouraging to see that mature T-ALL cells can be induced to undergo apoptosis by TCR activation, using the gene signature for negative selection that is reminiscent in these cells. Subsequently, when assessing the potential of novel anticancer therapies, it is necessary to also assess the importance of cell and disease, as TCR signaling supports oncogenesis in PTCL whereas it appears to have an anti-oncogenic effect in T-ALL.^413^

## Applications of TCR-based immunotherapy

The emergence of T cell-based immunotherapy has revolutionized the understanding of the role of T cells in mitigating a wide variety of diseases, including viral, autoimmune, and malignant diseases. TCR engineering has provided a compelling approach to fight cancer, disrupting the immuno-oncology research field and introducing a new class of impressive cancer immuno-therapeutic strategies, including adoptive cellular therapy (ACT), checkpoint blockade, tumor microenvironment (TME) regulation, and cancer therapeutic vaccines.

### Adoptive T cell transfer therapy

Experimental research in T cells adoptive transfer has revealed the superior capabilities of T cells to identify tumor antigens and to harness the immune system, contributing to anti-tumor activity.^418^ This type of therapy was first demonstrated clinically by Southam et al.^419^ in 1966 when patients with unresectable cancer displayed tumor regression upon co-transplantation with patient-derived leukocytes and autologous tumor cells. Although this strategy has been successfully applied, adoptive T cell transfer has not been generalized widely due to the fact that the number of infiltrated T cells was insufficient to exert a full potential of the anti-tumor activity or to boost the body’s immune response against cancer. In addition, the validated immune response in patients receiving this type of therapy was found to be cancer-type and patient-dependent.^420,421^ Therefore, the engineering of T cells has provided an effective alternative to activate and expand T cells ex vivo with defined specificity against tumor antigens. In this context, TCR-engineered lymphocytes have garnered considerable attention over the past decade, offering significant curative outcomes in patients with cancer. Because tumor cells downregulate MHC molecules, also known as HLA, this posed a challenge for proper T cell response directed against tumor antigen presentation resulting in immune tolerance.^422^ However, the development of synthetic chimeric antigen receptors (CARs) has overcome this challenge by redirecting T cell specificity to recognize and lyse tumor antigens on the surface of the malignant cell independently of MHC molecules.^423^

To date, different types of ACTs have been developed, including TCR engineered T cell therapy (TCR-T), tumor-infiltrating lymphocytes (TILs), and CAR T therapy.^424–427^ These strategies allow the fast entry of T cell-receptor-based immunotherapies to clinical trials with encouraging clinical outcomes.

### TILs therapy

TILs were the first classical attempt for ACT in which infiltrating T cells are isolated from the tumor mass and then expanded ex vivo, activated, and subsequently reinfused into the patient. Several reports have shown that TILs therapy induced a significant durable response in melanoma, including in patients resistant to immune-checkpoint blockade (ICB), as well as objective response in different types of cancers, such as gastrointestinal, colon, and breast cancers.^428–432^ However, this strategy has been hindered by limited access to solid tumors localized at restricted areas or that have non-resectable metastases as well as long ex vivo processing time and insufficient anti-tumor immunity.^433^ Completed clinical trials which used TILs-based therapy are summarized briefly in Table 1.

**Table 1:** Completed clinical trials using TILs-based immunotherapy

Cancer type/conditions	Study title	Study type/phase	Intervention/treatment	Status	NCT number
Metastatic ovarian cancer	TIL therapy in combination with checkpoint inhibitors for metastatic ovarian cancer	Interventional; Phase I and II	TILs in combination with checkpoint inhibitors	Completed	NCT03287674
Metastatic melanoma	Peginterferon and TIL therapy for metastatic melanoma	Interventional; Phase I and II	TILs infusion including lymphodepleting chemotherapy and interleukin-2	Completed	NCT02379195
Metastatic melanoma	Vemurafenib and TIL therapy for metastatic melanoma	Interventional; open-label Phase I and II	T cell Therapy in combination with Vemurafenib	Completed	NCT02354690
Multiple myeloma	Trial of activated marrow infiltrating lymphocytes alone or in conjunction with an allogeneic granulocyte macrophage colony-stimulating factor (GM-CSF)-based myeloma cellular vaccine in the autologous transplant setting in multiple myeloma	Interventional; Phase II	Activated marrow infiltrating lymphocytes alone or in conjunction with an allogeneic GM-CSF vaccine	Completed	NCT01045460
Multiple myeloma and plasma cell neoplasm	Activated white blood cells with ASCT for newly diagnosed multiple myeloma	Interventional; Phase I and II	Activated marrow infiltrating lymphocytes	Completed	NCT00566098
Melanoma	Phase II study of short-term cultured anti-tumor autologous lymphocytes after lymphocyte-depleting chemotherapy in metastatic melanoma	Interventional; Phase II	Cultured anti-tumor autologous lymphocytes following a lymphocyte depletion	Completed	NCT00513604
Melanoma	Phase II study of metastatic melanoma with lymphodepleting conditioning and infusion of anti-MART-1 F5 TCR-gene-engineered lymphocytes	Interventional; Phase II	Lymphodepletion followed by infusion of anti-MART-1 F5 TCR-gene engineered lymphocytes	Completed	NCT00509288
Melanoma neoplasm metastasis	Lymphocyte re-infusion during immune suppression to treat metastatic melanoma	Interventional; Phase II	Lymphocyte re-infusion during immune suppression	Completed	NCT00001832

### TCR-T therapy

**Fig. 5:**
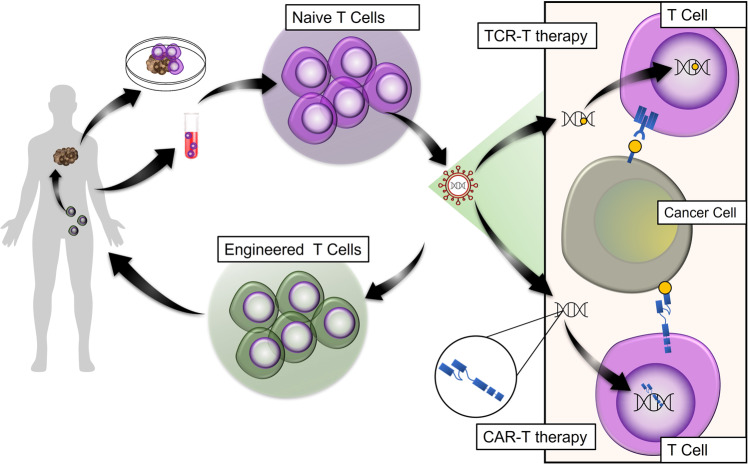
Schematic illustration of TCR-based immunotherapy. T cells are isolated from the patient’s cancer tissue or peripheral blood and genetically modified by retroviral transduction to express antigen-specific TCR or CAR on T cells. Cells are then expanded ex vivo until sufficient cell numbers are achieved and reinfused into the patient’s body, where they can fight cancer cells.

TCR-T therapy was developed to overcome some drawbacks of TILs therapy. This strategy utilizes the same principle as TILs but with genetic modification through retroviral transduction of TCRs to recognize tumor-specific antigens via MHC (Fig. 5). Despite the success of this therapeutic approach, the specificity remains challenging because tumors usually escape such attacks by downregulating MHC. The first clinical outcomes of TCR-T therapy were reported in 2006 when Morgan et al.^434^ demonstrated durable response among patients with melanoma after transducing autologous T cells with a TCR recognizing the melanocyte differentiation antigen (MART-1). Subsequent clinical trials in 2009 and 2014 confirmed TCR-mediated tumor regression in 30% and 69% of metastatic melanoma patients using MART-1 and gp100 TCR-engineered T cells, respectively.^435,436^ Parallel clinical responses were also documented using cancer-testis antigens, such as MAGE-A3 and NY-ESO-1. Robbins et al.^437^ observed that 6 out of 11 patients with synovial cell sarcoma and 5 out of 11 patients with melanoma treated with TCR targeting NY-ESO-1 antigen displayed objective responses. Moreover, targeting NY-ESO-1 antigen in multiple myeloma (MM) using TCR-T therapy has achieved similar robust clinical responses.^438^ These clinical data suggest that TCR-T therapy can potentially harness the immune system to target and eliminate cancer cells. Although the most commonly reported clinical outcomes were from clinical trials targeting melanoma, other clinical trials have started to introduce this type of therapy more frequently in other solid tumors. This is based on the fact that melanoma incidence has been increasing over the past few years more than other cancers.^439,440^ Furthermore, melanoma lesions are relatively accessible compared to other solid tumors; therefore, the means for ex vivo expansion of T cells can be readily available, making melanoma one of the best models for immuno-oncology not only in therapy but also in research purposes. A list of current active clinical trials using TCR-T is summarized in Table 2.

**Table 2:** Current active clinical trials using TCR-T cell-based immunotherapy

Cancer type/conditions	Study title	Study type/phase	Intervention/treatment	Status	NCT number
Recurrent hepatocellular carcinoma	TCR-redirected t cell treatment in patients with recurrent HBV-related hepatocellular carcinoma post liver transplantation	Interventional; Phase I (Open Label)	Biological: TCR-T cells by IV infusion	Active not recruiting	NCT04677088
Hematological malignancies	HA-1H TCR-T cell for relapsed/persistent hematologic malignancies after allogeneic stem cell transplantation	Interventional; Phase I	HLA-A* 02:01 restricted, HA-1H T cell receptor (TCR) transduced patient-derived T cell (MDG1021) immunotherapy	Active not recruiting	NCT04464889
Recurrent or refractory ovarian cancer	Genetically modified T cells and decitabine in treating patients with recurrent or refractory ovarian, primary peritoneal, or fallopian tube cancer	Interventional; non-randomized, open-label Phase I	Adoptive transfer of NY-ESO-1 TCR-engineered autologous T cells in combination with decitabine	Active not recruiting	NCT03017131
Melanoma, ovarian, and peritoneal carcinomas	Gene-modified T cells with or without decitabine in treating patients with advanced malignancies expressing NYESO-1	Interventional; Phase I and IIa	Autologous NY-ESO-1 TCR/dnTGFbetaRII transgenic T cells	Active not recruiting	NCT02650986
Non-small cell lung cancer or mesothelioma	Genetically Modified T Cells in treating patients with stage III-IV non-small cell lung cancer or mesothelioma	Interventional non-randomized; Phase I and II	Autologous WT1-TCRc4 Gene transduced CD8-positive Tcm/Tn Lymphocytes	Active not recruiting	NCT02408016
Metastatic solid tumors	T cell receptor immunotherapy targeting NY-ESO-1 for patients With NY-ESO-1 expressing cancer	Interventional; Phase II	Infusion of anti-NYESO-1 murine TCR-gene engineered lymphocytes	Competed	NCT01967823
Ovarian cancer	CT antigen TCR-redirected T cells for ovarian cancer	Interventional; Phase I and IIa, Open Label	Infusion with NYESO-1 (C259) transduced autologous T cells	Competed	NCT01567891
Malignant gliomas	CAR T cell receptor immunotherapy targeting EGFRvIII for patients with malignant gliomas expressing EGFRvIII	Interventional; Phase I and II	Administering T cells expressing anti-EGFRvIII CAR TCR	Completed	NCT01454596
Multiple myeloma	Redirected auto T cells for advanced myeloma	Interventional; Phase I/IIa	Autologous genetically modified T cells transduced to express the high-affinity NY-ESO-1c259 TCR in HLA-A2+ subjects	Completed	NCT01352286
Malignant melanoma	Study to assess the tolerability of a bispecific targeted biologic IMCgp100 in malignant melanoma	Interventional; Phase I	Monoclonal T cell receptor Anti-CD3 scFv fusion protein, IMCgp100	Completed	NCT01211262
Melanoma	Radiation, chemotherapy, vaccine and anti-MART-1 and anti-gp100 cells for patients with metastatic melanoma	Interventional; Phase II randomized open label	Infusion of anti-Mart-1 and anti-gp100 TCR-gene engineered lymphocytes and peptide vaccines	Completed	NCT00923195
Melanoma	Phase II study of metastatic melanoma with lymphodepleting conditioning and infusion of anti-MART-1 F5 TCR-gene engineered lymphocytes	Interventional; Phase II non-randomized open label	Infusion of anti-MART-1 F5 TCR-gene engineered lymphocytes	Completed	NCT00509288
Metastatic cancers	Phase II study of metastatic cancer that overexpresses P53 using lymphodepleting conditioning followed by infusion of anti-P53 TCR-gene engineered lymphocytes	Interventional; Phase II, non-randomized open label	Infusion of anti-p53 T cell receptor (TCR)-gene engineered lymphocytes	Completed	NCT00393029

### CAR T cell therapy

CAR T cell therapy is lauded as a major step in the development of personalized cancer treatment. The patient’s own T cells are collected by leukapheresis (or peripheral blood) and genetically modified to express a synthetic receptor that binds a specific tumor antigen. These cells are then activated and expanded ex vivo and reinfused into the patient to target and attack cancer cells (Fig. 5). Unlike traditional T cells, CAR T cells recognize antigens independently of MHC presentation due to CAR’s unique structure, containing a transmembrane region with antigen-binding domain and intracellular signaling and co-signaling domains, allowing MHC-independent CAR T cells to bind to their target. Thus, CAR-T cell therapy can overcome cancer-mediated immune tolerance response. Unprecedented clinical response with a high remission rate has been observed using anti-CD19 CAR-T cell therapy in treating patients with B cell malignancies, including B cell acute lymphoblastic leukemia (B-ALL), chronic lymphocytic leukemia (CLL), and B cell non-Hodgkin lymphoma (B-NHL).^441–445^ In a phase II multicenter clinical trial, anti-CD19 CAR-T therapy was conducted on patients with refractory B cell lymphomas where 82% of patients displayed significant tumor regression and 54% showed complete response rate.^446^ Moreover, a systematic review and meta-analysis of all published clinical trials conducted by Irbaz Bin Riaz et al. to study the efficacy and safety of anti-CD19 and anti-CD20 CAR-T therapy for B cell hematologic malignancies showed that, among 16 eligible studies, the overall response rate was 61% with complete and partial responses of 42% and 19% respectively. Another clinical trial aimed at determining long-term follow-up of anti-CD19 CAR- T cell therapy has reported the longest durable remission in patients with B cell lymphoma—up to 113 months after treatment—suggesting that anti-CD19 CAR T cells may be curative for B cell lymphoma.^447^ The success of anti-CD19 CAR-T therapy could be related to the high expression of CD19 in some B cell malignancies and its specificity to the B cell lineage. However, clinical studies showed that the loss of CD19 antigen following treatment is a common cause of disease relapse.^448^ Thus, anti-CD22 CAR-T has emerged as a potential alternative to anti-CD19 CAR-T therapy. Clinical trials have shown that anti-CD22 CAR-T cells could overcome resistance mediated by anti-CD19 CAR-T cell immunotherapy in patients with B-ALL.^449^

B cell maturation antigen (BCMA/CD269) targeted therapy has emerged as a promising target for CAR-T cell immunotherapy in multiple myeloma (MM). Although this target is still under investigation, clinical trials phase I exhibited parallel safety and toxicity profiles and suggest its clinical activity against MM.^450–452^ In addition, CAR-T has been shown to target prostate cancer through directing CAR-T cells against prostate-specific membrane antigen (PSMA) and displayed an acceptable safety and efficacy profile.^453^ In a phase I clinical study, Yao Wang et al.^454^ targeted patients with CD133-positive and late-stage metastasis malignancies by CAR-T cells in which three patients exhibited partial remission and 14 achieved stable disease.

Despite the success of CAR-T therapy in hematological malignancies, solid tumors have introduced a greater challenge owing to their immunosuppressive microenvironment. While CAR-T therapy has rendered the TME more immunogenic, CAR-T has generated a significant toxic profile; for example, cytokine release syndrome, neurotoxicity, therapy-related mortality, and manufacturing issues have complicated CAR-T cell therapy for solid tumors.^455–458^ Efforts to overcome these challenges to generate a more favorable toxicity with CAR-T cell therapy are ongoing.

## Immune-checkpoint blockade

A growing body of evidence indicates that peripheral T cell tolerance is an essential factor of the specific immune response to tumor cells. The low cytotoxic capabilities of T cells may be related to the high expression levels of a number of inhibitory molecules including Cytotoxic T lymphocyte antigen 4 (CTLA4) and programmed cell death 1 (PD1). These evolutionarily conserved negative T cell activation regulators act as checkpoint molecules. CTLA4 and PD1 are highly expressed by various types of cancers, and their binding to their respective ligands contribute to T cell functional impairment, which fails to elicit the required immunity against minimal residual disease, and thereby play an important role in cancer recurrence.^459^ The discovery of immune-checkpoint’s role in cancer has changed the paradigm of cancer therapeutics and added immunotherapy to the list of common three cancer pillars including surgery, targeted therapy, radiotherapy, and chemotherapy.

PD1 and CTLA4 are the most extensively studied immune-checkpoint negative regulators due to their prominent role in fine-tuning tumor-infiltrating T cells. Targeting PD1 and its ligand programmed death-ligand 1 (PD-L1), as well as CTLA4, have gained immense attention after they had shown an unprecedented objective and durable responses across many clinical trials in a subset of patients of metastatic and unresectable cancers leading to different lines of FDA-approved immune-checkpoint inhibitors, and thereby translating checkpoint blockade therapy into an integral part of clinical standard therapy.^460–466^ For example, PD-1/PDL-1 inhibitors are now considered as a first-line treatment for patients with melanoma after they have demonstrated a significant increase in overall survival compared to dacarbazine chemotherapy.^467^ PD-1 inhibitors such as nivolumab and pembrolizumab have shown clinical efficacy in several lines of solid and hematological neoplasms including non-small-cell lung (NSCLC), bladder, pancreatic, follicular B cell, and non-Hodgkin lymphoma.^468,469^ In addition, it is worth noting that most of the observed effects were correlated with the extent of tumor-infiltrating T cells. Furthermore, in NSCLC, pembrolizumab displayed an improved objective response in patients harboring a high nonsynonymous mutational burden due to a defect in the DNA repair pathway, molecular smoking signature, and higher neoantigen burden.^470^ In a clinical study conducted to evaluate the correlation between immune cell infiltration and the clinical outcomes in pancreatic ductal adenocarcinoma, with respect to immune-checkpoint molecules, Rong Liu et al.^471^ found that increase infiltration of PD-1-positive T cells is associated with favorable patient’s prognosis and overall survival. Moreover, patients with melanoma who respond to anti-PD-1 therapy displayed increased intratumoral CD8+ T cells which were associated with tumor regression.^472^ Caroline Robert et al.^473^ reported significantly longer overall survival in patients with previously untreated metastatic melanoma using combination therapy of ipilimumab and dacarbazine. Previous reports also indicated that ipilimumab and nivolumab combination therapy exhibited a significant survival benefit in patients with advanced renal cell carcinoma and metastatic melanoma.^474,475^ Although this line of evidence supports the beneficial role of checkpoint blockade combination therapy, it increases the risk of drug-induced toxicity and therefore should be evaluated with caution.

## Targeting the TME

**Fig. 6:**
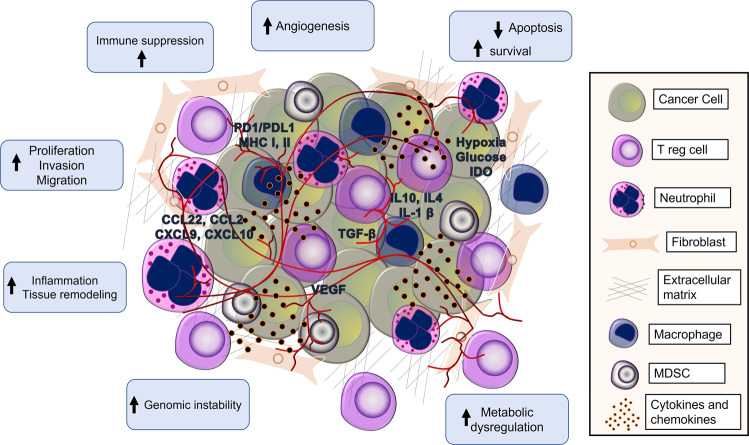
Schematic representation of the tumor microenvironment (TME). The immunosuppressive microenvironment induced by cancer-associated stromal cells modulates cancer progression and therapy resistance. Infiltration of immune cells, such as T reg cells, N2 neutrophils, tumor-associated macrophages, MDSC cells, the transformation of malignant fibroblasts, release of pro-inflammatory cytokines and chemokines, dysregulated vasculature and extracellular matrix remodeling, overexpression of negative immune-checkpoint regulators, metabolic status of the tumor including O_2_ and nutrients deprivation, the genetic composition of the tumor cells, all this heterogeneous ecosystem of the TME contributes to the tumor therapy resistance.

One of the most common challenges in TCR-based immunotherapy is TME. The TME promotes an immunosuppressive nest through: (1) tumor tissue remodeling by regulation of ECM and inhibition of T cells migration^476^; (2) recruitment of tumor-associated stromal cells, such as T regulatory cells (T reg), myeloid-derived suppressor cells, and tumor-associated fibroblasts^477^; (3) production of immunosuppressive cytokines and chemokines, such as TGFβ, IL-10, indoleamine 2,3-deoxygenase (IDO), CCL2, and CCL22 (ref. ^478^); (4) the metabolic state of the tumor tissue that is tightly regulated by oxygen, amino acids, and glucose levels^479^; (5) expression of ligands that activate inhibitory receptors, such as CTLA4 or PD-1 (ref. ^476^); (6) epigenetic regulation of the tumor stromal cells—for example, CXCL9 and CXCL10 silencing caused by DNA methylation inhibit T cells homing, thereby cause resistance to immune-checkpoint drugs^480^; (7) promoting T cells anergy, a process of self-inhibition that results from TCR activation in low levels or absence of appropriate co-stimulation^481^ (Fig. 6). Taken altogether, these mechanisms reflect the high degree of the tumor heterogeneity that is implicated in tumor evasion, prevent tumor destruction by T cells, and contribute to the development of dysfunctional antitumor immune responses.

Several immuno-modulatory strategies have been proposed to target the TME by which the T cell-immune response can be reactivated. For example, it has been found that targeting CTLA4 by ipilimumab reduced the number of infiltrating T reg cells.^482,483^ However, it is still unclear whether the predominant effect observed by ipilimumab therapy is due to T reg cells depletion or due to effector T cells (eff T cells) infiltration. Studies that demonstrated modest clinical outcomes of ipilimumab in cancer patients support the notion of depleting intratumoral T reg cells^484,485^ whereas other studies failed to observe such findings because tremelimumab, an IgG2 isotype CTLA4 antibody with minimal potential antibody-dependent cytotoxicity, displayed similar clinical activity as ipilimumab, suggesting that Treg depletion is not crucial for CTLA4 antibodies-mediated cancer regression.^486^ It is believed that CTLA4-antibodies-mediated depletion of Treg cells in tumors is regulated to a large extent by the host Fc receptor polymorphisms and the availability of effectors of antibody-dependent cellular cytotoxicity in the TME.^487^ Thus, engineered CTLA4 antibodies with optimized Fc receptors to selectively deplete T reg cells represent an interesting approach towards developing specific anti-tumor immunity.

Lymphocyte-activation gene 3 (LAG-3) is a cell surface molecule expressed on activated eff T and Treg cells in the TME. Binding LAG-3 to its ligand MHC class II mediates suppression of eff T cell activity and upregulation of Treg activity, contributing to the immune tolerance of the TME.^488^ Antibodies directed against LAG-3 have shown a modest impact on the clinical outcomes in patients with renal, pancreatic, and metastatic breast cancers.^489^ The evaluation of LAG-3 alone or in combination with other immune checkpoints in clinical trials is ongoing.

Another avenue to manipulate the TME is by promoting T cells response while binding other immune effectors simultaneously. In this context, bispecific antibodies have emerged as a successful strategy to target co-stimulatory and co-inhibitory molecules, including PD-L1 and LAG-3 to dampen the suppressive TME. Bispecific blinatumomab, which binds CD19 on the tumor cells and CD3 on the T cells, showed promising success in patients with acute lymphoblastic leukemia.^490^

Several signaling pathways have been implicated in tumor-associated molecular alterations that contribute to the immune suppression of the TME, such as Kirsten rat sarcoma viral oncogene (KRAS), focal adhesion kinase (FAK), and Janus kinase-1/2 (JAK1/2). KRAS and FAK signaling promote the recruitment of myeloid suppressor and T reg cells through the granulocyte–monocyte colony-stimulating factor (GM-CSF) in KRAS-associated cancers and CCL5 in squamous cell carcinoma, leading to T cell exhaustion.^491,492^ FAK has also been reported to mediate SRC kinase negative TCR regulation following T cell activation.^493^ Furthermore, JAK1 and JAK2 loss-of-function mutations were found to be correlated to acquired PD-1 blockade resistance in melanoma.^494^ Thus, these data suggest that aberrant signaling pathways are critical regulators of the TME, and targeting these pathways using TCR-based immunotherapy with selective pathway inhibitors might provide a rationale for a combinatorial approach and could overcome the immune activation resistance posed by the TME.

## Cancer vaccines

Cancer therapeutic vaccines have become an attractive tool to specifically direct T cell response towards tumor cells. Patients with cancer are exposed to tumor antigens in which T cells get activated, amplified, and elicit a tumor-directed immune response that can induce long-lasting memory T cells, mediating durable clinical responses. While most of the previous cancer vaccines utilized tumor-associated antigens (TAA), the currently developed approach is based on tumor-specific antigens (TSA), known as neoantigens, which result from somatic cancer cell mutations. Unlike TAAs, neoantigens are exclusively expressed by cancer cells with high immunogenicity without being negatively subjected to central or peripheral tolerance, and therefore they elicit specific tumor T cell immune response and prevent “off-target” that can damage the healthy tissues.^495^ Moreover, the advancement of newly emerging technologies such as next-generation sequencing and mass spectrometry-based algorithms have accelerated the translational and manufacturing aspects of vaccinomics and identified different cancer neoantigens for personalized immunotherapy, meaning it can be tailored individually to each cancer patient.

Several reports showed that TCR-based therapy combined with neoantigen vaccines provoke an efficient antitumor response. For example, neoantigen-reactive T cells have been found to express a high amount of PD-1 following neoantigen vaccines.^496–498^ In a study conducted by Patrick A. Ott et al., vaccination with neoantigens was associated with neoantigen-specific T cell expansion and induction of polyfunctional CD4^+^ and CD8^+^ T cells, which targeted 58 out of 97 unique neoantigens in melanoma patients. Moreover, four out of six patients were recurrence-free at 25 months after vaccination while the two with cancer recurrence were subjected to subsequent anti-PD-1 therapy and exhibited a complete cancer regression.^499^ In a phase Ib clinical trial, Keskin et al.^500^ demonstrated that neoantigen-specific infiltrating T cells express co-inhibitory receptors following cancer vaccination in glioblastoma patients, providing a rationale for neoantigen vaccines and immune-checkpoint blockade combination therapy. One study which used autologous tumor lysate-dendritic cell vaccine generated neoantigen-specific T cell responses following the vaccination in patients with ovarian carcinoma.^501^ The use of peptide vaccines and dendritic cells along with TCR-T therapy has also been reported. Anti-melanoma antigens recognized by T cells (MART-1) and anti-glycoprotein (GP-100) peptide vaccines have been shown to stimulate the immune cells and induce MART-1 and GP-100 specific TCR-T cells in patients with metastatic melanoma.^434,502^ Other clinical trials combining neoantigen vaccines with different cancer immunotherapies are summarized elsewhere.^503^ These clinical data indicate that cancer vaccines in combination with TCR-based immunotherapies could potentially boost the immune system to eradicate cancer cells while leaving behind long-lasting immune protection against cancer recurrence.

## Concluding remarks

The breakthrough made by TCR-based therapeutic applications is rapidly transforming the paradigm of immunotherapies. PD-1 and CTLA4 are the most extensively studied immune-checkpoint negative regulators due to their prominent role in fine-tuning tumor-infiltrating T cells. However, cancer cells exploit this negative regulation and escape from the immune system surveillance. Moreover, due to a wide variety of PD-1 or PD-L1 expression among different types of cancers, not all patients are eligible to undergo this type of treatment. On the other hand, although CAR-T therapy has emerged as a potential strategy to target hematological malignancies, this type of therapy is still hindered by the challenges posed by the TME heterogeneity in solid tumors and by the accompanied therapy-related toxicities. While TCR-T cell therapy has provided impressive clinical results, given its ability to target solid tumors, incorporating cancer vaccines, and recognition of intracellular antigens, the development and proliferation of such therapy encounters numerous obstacles, such as manufacturing issues, tailoring treatment for each patient based on identified genetic mutations, and the tumor immunogenicity. However, we believe that the development of the next generation of TCR-based therapy will overcome these dilemmas, and more groundbreaking applications for cancer immunotherapy are expected to be revealed in the near future.
